# Psychosocial work exposures and health outcomes: a meta-review of 72 literature reviews with meta-analysis

**DOI:** 10.5271/sjweh.3968

**Published:** 2021-09-30

**Authors:** Isabelle Niedhammer, Sandrine Bertrais, Katrina Witt

**Affiliations:** INSERM, Univ Angers, Univ Rennes, EHESP, Irset (Institut de recherche en santé, environnement et travail) - UMR_S 1085, ESTER Team, Angers, France; Orygen, the National Centre of Excellence in Youth Mental Health and the Centre for Youth Mental Health, The University of Melbourne, ­Melbourne, Australia

**Keywords:** pooled estimate, systematic literature review

## Abstract

**Objective:**

This meta-review aimed to present all available quantitative pooled estimates for the associations between psychosocial work exposures and health outcomes using a systematic literature review of literature reviews with meta-analysis.

**Methods:**

A systematic review of the literature from 2000 to 2020 was conducted using PubMed, Web of Science, Scopus, and PsycINFO databases following the PRISMA guidelines. All literature reviews and Individual-Participant Data (IPD)-Work consortium studies exploring an association between psychosocial work exposures and health outcomes and providing pooled estimates using meta-analysis were included. All types of psychosocial work exposures and health outcomes were studied. The quality of each included review was assessed.

**Results:**

A total of 72 reviews and IPD-Work consortium studies were included. These mainly focused on job strain as exposure and cardiovascular diseases and mental disorders as outcomes. The associations between psychosocial work factors and cardiovascular diseases and mental disorders were in general significant, and the magnitude of these associations was stronger for mental disorders than for cardiovascular diseases. Based on high-quality reviews, significant associations were found between job/high strain and long working hours as exposures and coronary heart diseases, (ischemic) stroke, and depression as outcomes. A few additional significant associations involved other exposures and health outcomes.

**Conclusions:**

The included reviews brought convincing findings on the associations of some psychosocial work factors with mental disorders and cardiovascular diseases. More research may be needed to explain these associations, explore other exposures and outcomes, and make progress towards determining the causality of the associations.

Psychosocial work exposures emerged in the area of occupational health epidemiology during the 1990s, the first studies being published in the 1980s (1–5) and some very rare studies in the 1970s (6). Since then, the literature has expanded considerably, making a synthesis of the literature timely. Psychosocial work factors are characterized by a multitude of exposures, which presents problems in summarizing the literature. A number of studies have investigated the factors of the job strain model, one of the first and widely used theoretical model, including decision latitude, psychological demands, job strain (combination of high demands and low latitude), and social support. However, psychosocial work factors embrace a much higher number of aspects, such as long working hours, job insecurity, effort–reward imbalance, but also more recently workplace bullying, organizational injustice, and work–family conflict, amongst others. In addition, this is not only the issue of exposure that is complicated but also the issue of outcome, as the diversity of outcomes also adds to complexity in the field.

A large amount of the literature has focused on the associations of psychosocial work exposures with mental disorders and cardiovascular diseases. Various other health outcomes have also been investigated, although less frequently, such as cardiovascular risk factors, behavioral risk factors, and more rarely other diseases. Psychosocial work factors may be expected to be associated with a large number of health outcomes, consequently for this reason too, a synthesis of the literature may also be difficult to achieve.

A substantial number of literature reviews have been published on specific associations between psychosocial work exposures and health outcomes in recent years. Four meta-reviews, based on literature reviews, have been published so far and summarized the evidence for two outcomes, cardiovascular diseases (7) and common mental health problems (8), and two exposures, workplace bullying (9) and long working hours (10). Three of these meta-reviews used a systematic procedure to synthesize information, and only one provided quantitative pooled estimates. Focusing on literature reviews with meta-analysis based on primary studies that have already been selected on the basis of well-defined criteria may be useful in order to summarize the literature in a quantitative way. Indeed, an additional problem may be the heterogeneity of the literature regarding the quality of the studies. In addition, given the inherent problems related to the study of the associations between psychosocial work exposures and health outcomes (such as residual confounding and reporting bias), causal inferences may be difficult to reach and a limited number of primary studies in the literature or pooled in a meta-analysis may not be enough to provide the level of evidence required. Finally, a state-of-the-art providing quantitative pooled estimates may be particularly helpful to other research topics such as those related to the estimation of fractions and costs attributable to psychosocial work exposures.

Our aim was therefore to perform a meta-review (ie, a systematic literature review of literature reviews with meta-analysis) on the associations between psychosocial work exposures and health outcomes and to report all available quantitative pooled estimates for each of these associations. As our aim was to provide a comprehensive review on the etiological effects of psychosocial work exposures, all psychosocial work factors and all health outcomes were included. We further investigated the significance, magnitude, precision, and consistency over time of the associations, and the differences between outcomes to provide more information about the specificity of the effects or on the contrary about the multiple effects of these exposures on health.

## Methods

### Search strategy

We systematically searched for published systematic literature reviews with meta-analysis using PubMed, Web of Science, Scopus, and PsycINFO databases from 1 January 2000 to 28 September 2020. Keywords were chosen to capture two criteria: firstly, the articles had to be a literature review with meta-analysis, and secondly, the reviews had to explore associations for any psychosocial work exposures. A list of keywords was therefore developed to cover these two criteria (Appendix 1, www.sjweh.fi/article/3968). The comprehensiveness of our search was checked using the reference lists of the included reviews. The meta-review was conducted according to the Preferred Reporting Items for Systematic Reviews and Meta-Analyses (PRISMA) guidelines (www.prisma-statement.org).

### Inclusion criteria, eligibility, and selection of reviews

Literature reviews were eligible for inclusion in this meta-review if: (i) keywords (Appendix 1) were present in the titles and/or abstracts, (ii) they were published in English, and (iii) they were published from 1 January 2000 to 28 September 2020. All psychosocial work exposures were included. However, factors related to time schedules, that may have an impact on health outcomes through disruption of circadian rhythms, such as night work or shift work or other atypical work schedules, were not considered. All health outcomes were included as far as they were related to symptoms, disorders, or diseases coded in the International Classification of Diseases, version 10 (ICD-10). Any behavioral disorders due to psychoactive substance use were also included, as well as any behavioral risk factors. However, we excluded outcomes that could not be linked to specific diseases or disorders, such as all-cause mortality, sickness absence, accident/injury, disability, self-reported health, well-being, or quality of life. Furthermore, as the objective was to study the etiological role of psychosocial work exposures, we excluded reviews that explored disease recurrence, chronicity, or exacerbations. In order to extrapolate the results as far as possible to the whole working population, the population had to be a general working population, or at least had to be as varied as possible in terms of occupations and work sectors, thus reviews focusing on one specific occupation or work sector were excluded. The reviews had to include a meta-analysis. We also included the studies of the IPD-Work (individual-participant data meta-analysis in working populations) consortium as these studies were highly relevant because they included large samples from a range of countries and results from meta-analysis even if not always based on a literature review (11). Indeed, this consortium published two types of papers: the first type including unpublished pooled analyses of IPD-Work cohorts only (called IPD-Work consortium studies without literature review in our text) and the second type including both literature reviews and unpublished pooled analyses of IPD-Work cohorts (called IPD-Work consortium studies with literature review in our text). Both types were included in our meta-review. For simplicity, we called ‘reviews’ all publications included in our meta-review. Finally, the reviews had to include a meta-analysis that provided a quantitative pooled estimate for the exposure–outcome association, ie, relative risk (RR), hazard ratio, or odds ratio. If the presentation of the results by the authors led to inverse pooled estimates, then the estimates were reversed (for example, estimate for high support instead of low support). We retained pooled estimates adjusted for gender, age, and socioeconomic status (SES), or if not available the closest minimally adjustment, to make comparison possible as far as possible. If it was possible, adjustment for behavioral risk factors was not retained in the study of cardiovascular diseases, as these behavioral risk factors may be mediators in the studied exposure–outcome associations. The estimates based on one primary study only were not retained. The retained pooled estimates were presented as ‘main results’ in the data extraction.

**Figure 1:**
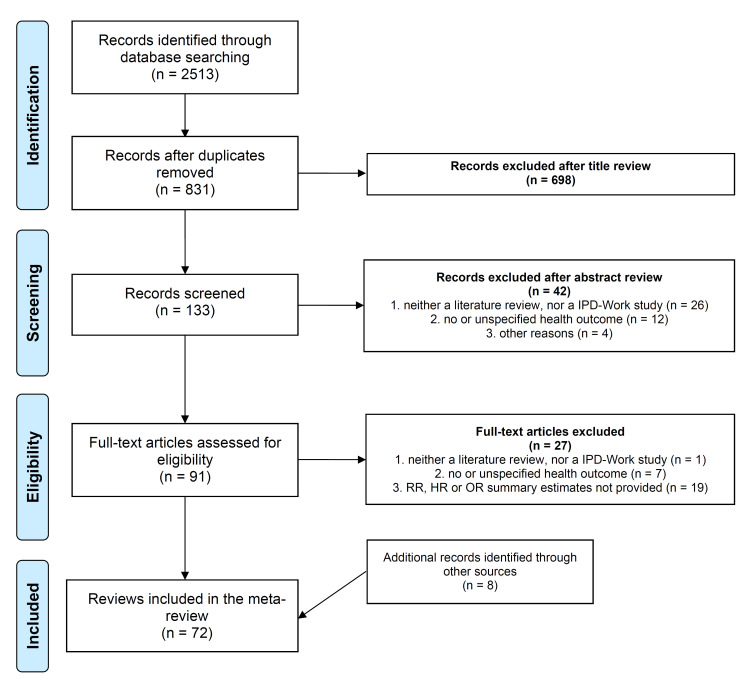
Selection process of reviews.

Two of the authors (IN and SB) independently conducted the systematic search, screening and selection. In case of inconsistencies, classification mismatches were discussed and resolved by consensus. Figure 1 presents the selection process.

### Data extraction

A standardized form was used to collect information about all included reviews and two of the authors (IN and SB) independently extracted data, with any discrepancies resolved by consensus. Extracted data were presented in supplementary table S1.

A number of instruments have been designed to assess the quality of systematic literature reviews, especially those including randomized studies. However, such instruments based on criteria checklists may not be sufficient to draw a conclusion on evidence (12). Indeed, these checklists generally do not collect enough information about the methodological aspects of the non-randomized and epidemiologic studies. Furthermore, as we included systematic reviews as well as IPD-Work consortium studies without literature review, the use of these checklists would have been irrelevant for these studies. Consequently, inspired by some checklists such as AMSTAR 2 (13), we elaborated a list of critical points including methodological criteria and assessed each review and IPD-Work consortium study. Quality scores were calculated ranging from 0–16 with higher scores indicating higher quality. For IPD-Work consortium studies without literature review, a weighted score was calculated (Appendix 2). The results for quality assessment were presented in supplementary table S2.

The pooled estimates of the main results from the included reviews were presented using figures according to exposure, outcome, publication date, and quality score. Significance, magnitude, and precision of the associations were presented using pooled estimates and their 95% confidence interval (CI). If there was more than one pooled estimate for a given association, the results were studied for consistency according to publication date. In the figures, diseases or disorders were preferred to symptoms (eg, depression versus depressive symptoms), and main exposure dimensions were preferred to sub-dimensions (eg, support versus colleagues/supervisors support), when both were available. All information may be found in supplementary table S1.

## Results

Among the eligible articles for inclusion in the selection process, 72 reviews with meta-analysis were included in our meta-review (14–85) (figure 1). Supplementary table S3 presents the 27 excluded articles after fulltext reading and the reason for exclusion.

### Characteristics of the reviews included in the meta-review

Almost all included reviews were published after 2011. Among the 72 reviews, 47 (65%) were literature reviews with meta-analysis, 7 (10%) were IPD-Work consortium studies with literature review, and 18 (25%) were IPD-Work consortium studies without literature review. The reviews included an average of 20 primary studies (range 6–86).

The job strain model exposures were the most frequently studied exposures: job strain or high strain (the difference between these two exposures is related to the reference group: either low demands or high latitude or both for job strain, and low demands and high latitude, also called low strain, for high strain) (37 reviews, 51%), psychological demands and decision latitude (17 reviews each, 24%), and social support (13 reviews, 18%). Long working hours were the second most frequently studied exposure (23 reviews, 32%). Effort–reward imbalance was explored in 12 reviews (17%), and job insecurity or temporary employment in 11 reviews (15%). Workplace bullying or violence were studied in 5 reviews (7%) and organizational injustice in 5 reviews (7%) as well. There were 2 reviews on emotional demands and 2 on work-life imbalance. A number of reviews examined more than one exposure from different concepts or models (14 reviews, 19%).

The most frequently studied outcomes were cardiovascular diseases: coronary heart disease (CHD) (15 reviews, 21%), cardiovascular risk factors (14 reviews, 19%), stroke (5 reviews, 7%), and behavioral risk factors (5 reviews, 7%). Other or unspecified cardiovascular diseases were examined in 5 additional reviews (7%). A large number of reviews explored mental health outcomes: depression-related outcomes (9 reviews, 12.5%), sleep problems (6 reviews, 8%), anxiety or burnout (5 reviews), psychotropic medication use (2 reviews), and suicide-related outcomes (1 review). Unspecified common mental disorders, pregnancy outcomes, and musculoskeletal disorders (MSD) were studied in 5 reviews each. An additional 3 reviews focused on cancer and 1 on digestive diseases.

The quality was low for 22 reviews (score≤7), moderate for 30 reviews (score 8–11), and high for 20 reviews (score≥12) (supplementary table S2). The mean score was 9.7. The mean score was 8.3 for systematic reviews, 12 for IPD-Work consortium studies with literature review, and 12.7 for IPD-Work consortium studies without literature review. However, for this last group, as the quality score was assessed using 5 criteria instead of 8, the comparison may be difficult with the two other groups. Among the 54 reviews (the 18 IPD-Work consortium studies without literature review excluded), 39 (72%) followed guidelines. Prospective design was the retained study design to select the primary studies, alone (46 reviews, 64%) or combined with case–control studies (9 reviews, 12%). Adjustment for gender, age, and SES was used in 16 reviews (22%), or a close adjustment in 18 additional reviews (25%). Quality was assessed in 37 reviews among 54 (69%). Almost all reviews performed the meta-analysis using adequate statistical methods (63 reviews, 88%). Publication bias was studied in 36 reviews among 54 (67%), and 21 of them did not find this bias whereas 15 found this bias. Heterogeneity was explored in 58 reviews (81%), and 23 of them found low heterogeneity. Among 67 reviews that included both genders, 30 reviews provided no information about gender. Attention was given to gender in 37 reviews (55%), however, gender differences were not always statistically tested, limiting definitive conclusions (9 reviews). A set of 27 reviews (96%) concluded to the absence of gender differences in the exposure–outcome associations, and only one reached the opposite conclusion. When other subgroup differences were tested, the large majority of the reviews did not find differences for age (81%), region/country (79%), and SES (69%). When explored and tested (which was very rare), there were no differences in the associations according to study characteristics such as study quality, study design, adjustment, exposure and outcome measurement, and follow-up length in (almost) all reviews.

### Pooled estimates for each exposure–outcome association

Table 1 shows the availability of at least one pooled estimate for all exposure–outcome associations. This table includes 16 exposures and 38 outcomes, making a total of 608 exposure–outcome associations possible. In fact, at least one pooled estimate was available for only 119 exposure–outcome associations (ie, 20%).

**Table 1:** Summary table of all exposure-outcome associations studied in at least one of the included reviews. ✓=at least one available pooled estimate.

	Job strain	High strain	Low decision latitude	Psychological demands	Low social support	Long working hours	Effort-reward imbalance	Low reward	Job insecurity	Precarity	Temporary employment	Bullying	Violence	Organizational injustice	Emotional demands	Work-life imbalance
CHD	✓	✓	✓	✓		✓	✓		✓					✓		
Stroke	✓	✓				✓										
Ischemic stroke	✓	✓														
Hemorrhagic stroke	✓	✓														
Peripheral artery disease	✓															
Arterial fibrillation						✓										
Venous thromboembolism						✓										
Diabetes	✓		✓	✓	✓	✓			✓							
Obesity	✓					✓										
Physical inactivity	✓	✓				✓										
Smoking	✓					✓										
Alcohol intake	✓					✓									
Depression	✓		✓			✓	✓		✓			✓	✓			
Burnout			✓	✓	✓			✓	✓				✓	✓	✓	
Anxiety						✓			✓				✓			
Sleep problems	✓		✓	✓	✓	✓	✓					✓	✓	✓		✓
Suicide ideation	✓		✓	✓	✓		✓		✓							
Suicide			✓	✓	✓											
Psychotropics	✓		✓	✓	✓	✓	✓		✓	✓						✓
MSD (all regions)	✓		✓	✓	✓				✓		✓					
Low back pain	✓		✓	✓	✓				✓							
Neck/shoulder pain	✓		✓	✓	✓											
Upper extremity pain	✓		✓	✓	✓											
Lower extremity pain		✓		✓												
Miscarriage						✓										
Preterm delivery						✓										
Preeclampsia						✓										
Gestational hypertension						✓										
Small for gestational age						✓										
Low birth weight						✓										
Cancer (any)	✓					✓										
Colorectal cancer	✓					✓										
Lung cancer	✓					✓										
Breast cancer	✓					✓										
Prostate cancer	✓					✓										
Esophagus cancer	✓															
Crohn’s disease	✓															
Ulcerative colitis	✓															

**Figure 2:**
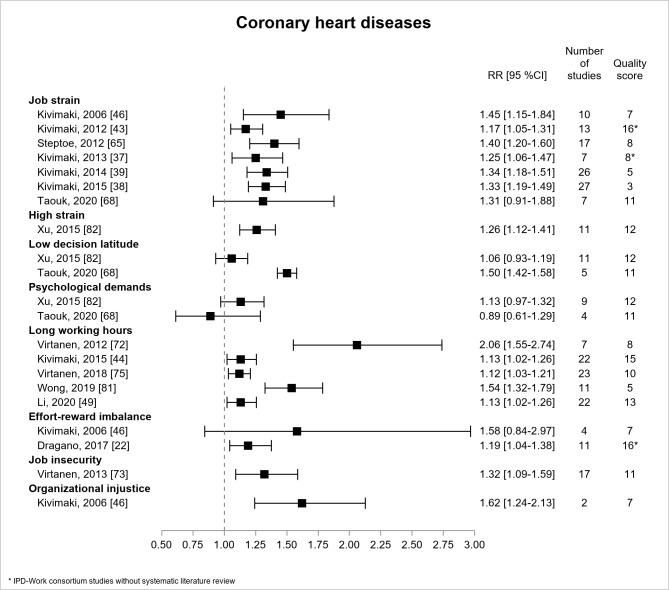
Pooled estimates for the associations between psychosocial work exposures and coronary heart diseases. * IPD-Work Consortium studies without systematic literature review.

Figure 2 presents the results for CHD. There were 15 included reviews (22, 37–39, 43, 44, 46, 49, 65, 68, 72, 73, 75, 81, 82). All were literature reviews except three IPD-Work consortium studies without literature review (22, 37, 43). All the estimates were significant for job/high strain and CHD, with increasing precision over time. The most conservative estimate (RR=1.17, 95% CI 1.05–1.31) (43), adjusted for gender, age, and SES, had a higher quality. One recent review (68) which investigated CHD mortality (and not CHD) provided a non-significant estimate, with the largest CI (lower precision). Among the five significant estimates for long working hours, three, based on prospective design, provided lower values of similar magnitude (RR=1.12 or 1.13) and had higher precision and higher quality (44, 49, 75). Among the two estimates for effort–reward imbalance, the most recent was significant and had greater precision (RR=1.19, 95% CI 1.04–1.38) and higher quality (22). The estimates for job insecurity (RR=1.32, 95% CI 1.09–1.59, moderate quality) (73) and organizational injustice (RR=1.62, 95% CI 1.24–2.13, low quality) (46) were significant, but displayed lower precision. Thus, the magnitude of the association was similar for job strain, long working hours, and effort-reward imbalance, and a little higher for job insecurity and organizational injustice, though more imprecise.

**Figure 3:**
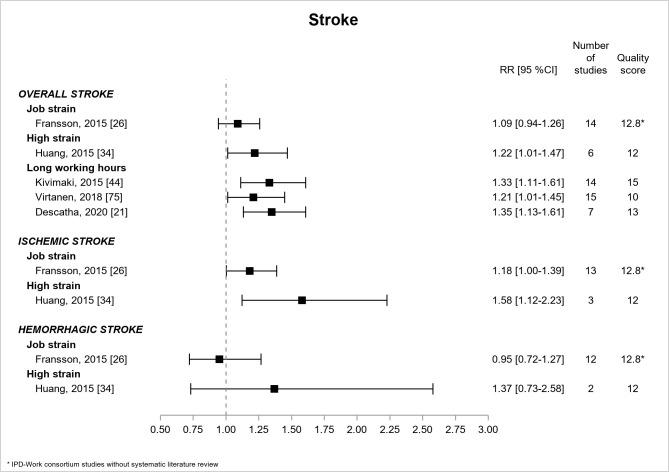
Pooled estimates for the associations between psychosocial work exposures and stroke. * IPD-Work Consortium studies without systematic literature review.

Figure 3 presents the results for stroke. Five papers (21, 26, 34, 44, 75) provided estimates that were based on literature reviews except one IPD-Work consortium study without literature review (26). For all, prospective design was a selection criterion and quality assessment was high, with one exception. High strain, but not job strain, was associated with overall stroke (RR=1.22, 95% CI 1.01–1.47) (34). The estimates were significant for ischemic stroke but not significant for hemorrhagic stroke. There were three significant estimates for the association of long working hours with overall stroke, the two higher quality estimates providing higher values of similar magnitude (RR of around 1.3) (21, 44).

Three IPD-Work consortium studies without literature review explored other cardiovascular diseases and reported significant associations between job strain and peripheral artery disease (high quality) (30), and long working hours and arterial fibrillation (high quality) (41) and venous thromboembolism (moderate quality) (40). The three RR ranged from 1.4–1.5.

**Figure 4:**
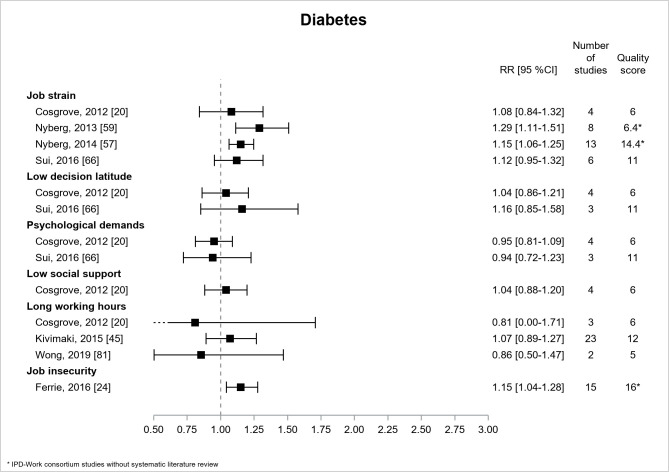
Pooled estimates for the associations between psychosocial work exposures and diabetes. * IPD-Work Consortium studies without systematic literature review.

Figure 4 presents the results for diabetes, which was the most frequently studied cardiovascular risk factor in 7 reviews (20, 24, 45, 57, 59, 66, 81), among which 3 were IPD-Work consortium studies without literature review (24, 57, 59). Most of the estimates were non-significant, and the three significant associations were reported by the 3 IPD-Work consortium studies without literature review. For job strain, there were 4 estimates, and only 2, of low or high quality, displayed significant associations with diabetes, the more conservative and more precise RR, of the two estimates, being 1.15, 95% CI 1.06-1.25 (high quality) (57). There was one significant association between job insecurity and diabetes (RR=1.15, 95% CI 1.04–1.28, high quality) (24).

**Figure 5:**
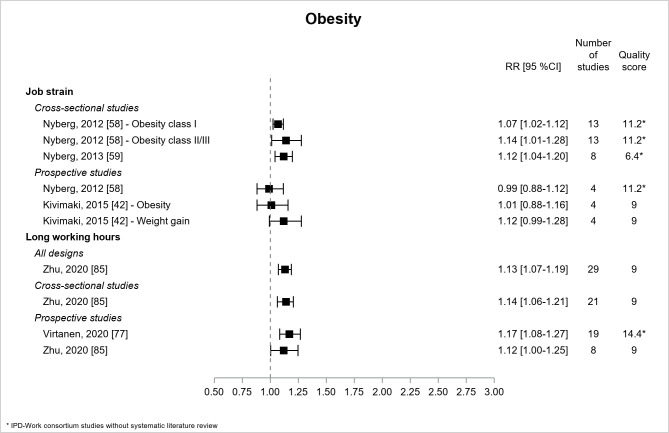
Pooled estimates for the associations between psychosocial work exposures and obesity. * IPD-Work Consortium studies without systematic literature review.

Figure 5 shows the results for obesity. There were 2 literature reviews (42, 85) and 3 IPD-Work consortium studies without literature review (58, 59, 77). Significant associations were observed between job strain and obesity using cross-sectional data but not using prospective data. The associations between long working hours and obesity were significant with all types of design (moderate/high quality), with 4 estimates of similar magnitude ranging from 1.12–1.17 (77, 85).

**Figure 6:**
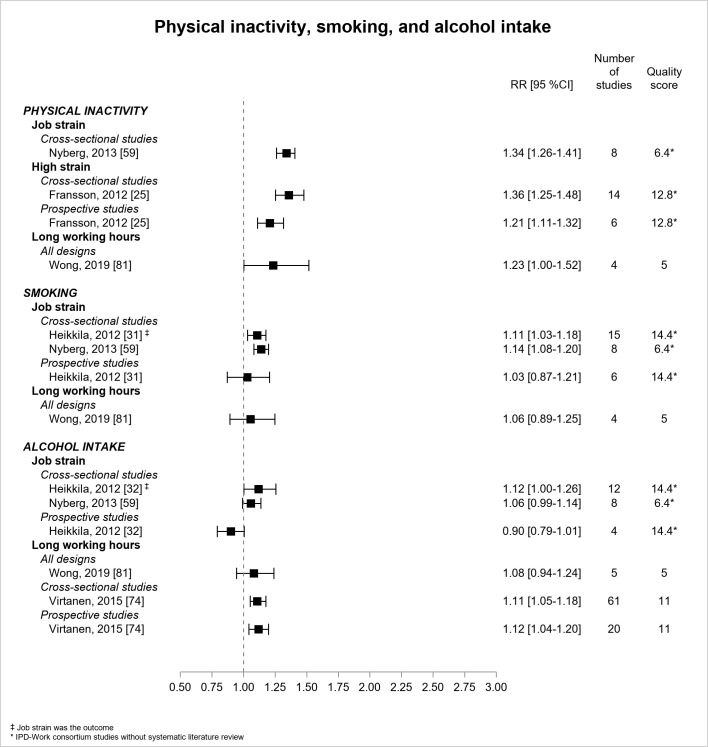
Pooled estimates for the associations between psychosocial work exposures and behavioral risk factors. ‡ Job strain was the outcome. * IPD-Work Consortium studies without systematic literature review.

Figure 6 presents the results for behavioral risk factors. Two IPD-Work consortium studies without literature review (25, 59) and one literature review (81) reported significant associations between job strain (59), high strain (25), long working hours (81) and physical inactivity. The estimate based on prospective design (RR=1.21, 95% CI 1.11–1.32) was more conservative than the estimate based on cross-sectional design for the association between high strain and physical inactivity (high quality) (25). Quality was low for the associations of job strain and long working hours with physical inactivity with significant RR of 1.34 (95% CI 1.26–1.41) (59) and 1.23 (95% CI 1.00–1.52) (81) respectively. Two IPD-Work consortium studies without literature review found a significant association between job strain and smoking using cross-sectional data, but not using prospective data. The association between long working hours and smoking was non-significant. Two reviews (74, 81) and two IPD-Work consortium studies without literature review (32, 59) examined alcohol intake. No association was observed between job strain and alcohol intake except one borderline significant based on cross-sectional design. Two literature reviews (74, 81) studied long working hours in association with alcohol intake, and one (moderate quality) reported two significant associations using either cross-sectional or prospective data with estimates of similar magnitude (RR of around 1.1) (74).

**Figure 7:**
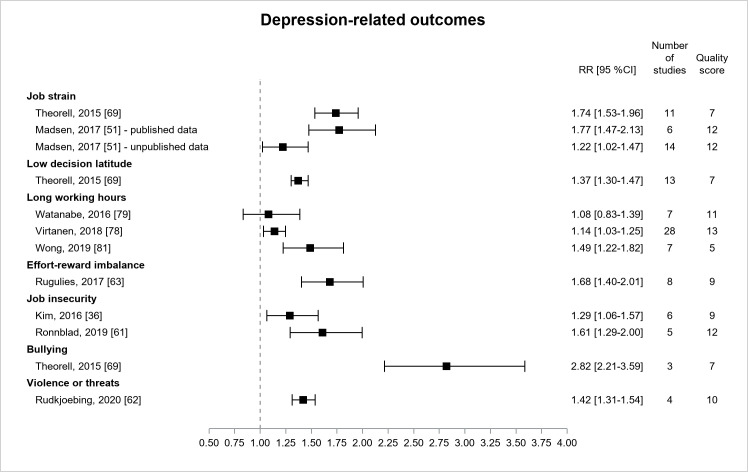
Pooled estimates for the associations between psychosocial work exposures and depression-related outcomes

Figure 7 presents the results for depression-related outcomes from nine reviews (36, 51, 61–63, 69, 78, 79, 81) examining depressive symptoms (36, 61, 63, 69, 78, 81), or more rarely, clinical/major depression (51, 62, 79) or hospital-treated clinical depression (51). All estimates were significant except one. All these estimates were derived from literature reviews of prospective primary studies, except one review of low quality (81), including cross-sectional design, that provided a stronger effect size of long working hours on depressive symptoms. The two first estimates (moderate/high quality) for long working hours were consistent in magnitude although the second one (RR=1.14, 95% CI 1.03–1.25) was significant and had greater precision and quality (78). Three estimates provided significant associations between job strain and depression with increasing quality from low to high. The two first estimates were consistent in terms of magnitude (RR ranging from 1.7–1.8) (51, 69). The third estimate was lower probably because the outcome was hospital-treated clinical depression (51). One estimate displayed a significant association between effort–reward imbalance and depressive symptoms (RR=1.68, 95% CI 1.40–2.01) (moderate quality) (63). There were two significant estimates for the association between job insecurity and depressive symptoms, and the most recent estimate had a higher quality (RR=1.61, 95% CI 1.29–2.00) (61). There was one significant estimate for the association between bullying and depressive symptoms, whose CI was large (RR=2.82, 95% CI 2.21–3.59) (low quality) (69). There was one significant estimate of lower magnitude for the association between physical violence and depression (RR=1.42, 95% CI 1.31–1.54) (moderate quality) (62). Thus, the associations of higher magnitude were found for job strain, effort-reward imbalance, job insecurity, and violence, and still more for bullying.

**Figure 8:**
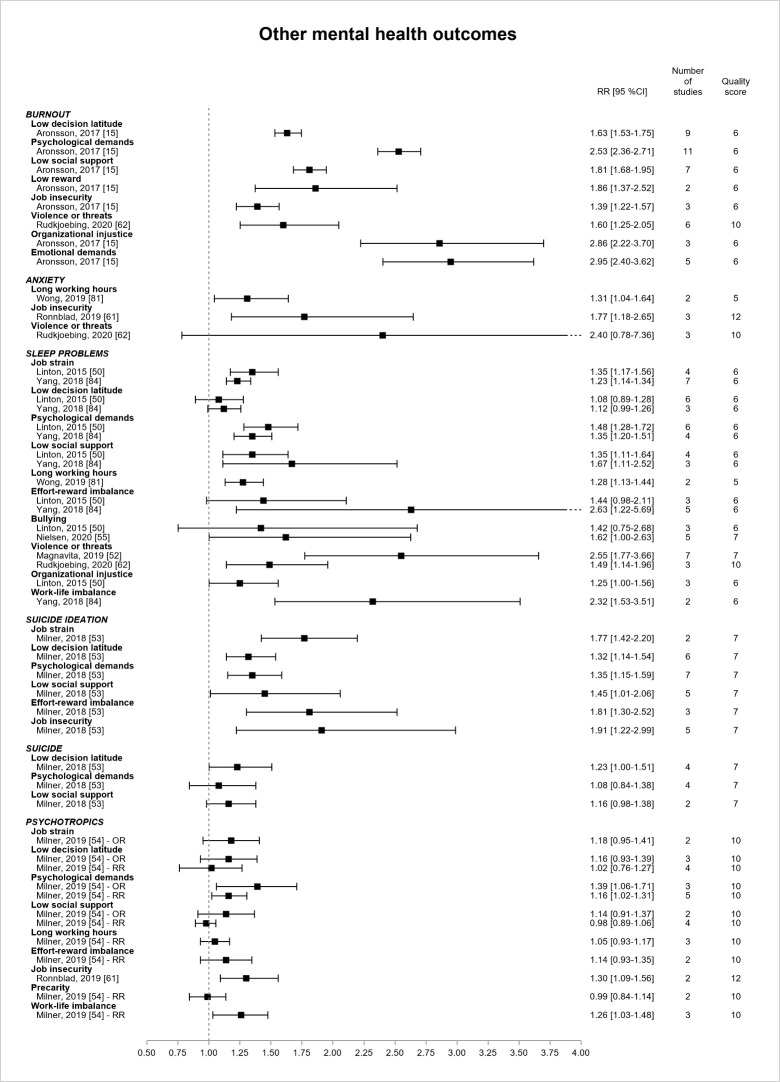
Pooled estimates for the associations between psychosocial work exposures and other mental health outcomes.

Figure 8 presents the results for the other mental health outcomes. Two literature reviews, of low or moderate quality, explored the associations between psychosocial work exposures and burnout (15, 62), only one was based on prospective design (15), and both provided estimates that were all significant. The magnitude of the association was larger for psychological demands, organizational injustice, and emotional demands (all RR>2.5). The precision was however low for reward, violence, injustice, and emotional demands. Three reviews reported estimates for anxiety symptoms (61, 62, 81), and two of them showed significant, though imprecise, associations with long working hours (RR=1.31, 95% CI 1.04–1.64, low quality) (81) and job insecurity (RR=1.77, 95% CI 1.18–2.65, high quality) (61). Six reviews provided estimates for sleep problems (low or moderate quality) (50, 52, 55, 62, 81, 84). They were all literature reviews but only two reviews selected prospective primary studies (50, 55). The estimates were significant for almost all exposures. The CI were however very large, except for the job strain model factors and long working hours. The RR ranged from 1.2–1.4 for job strain and long working hours. RR of higher magnitude (>2) were observed for effort–reward imbalance, violence, and work-life imbalance, but these estimates were very imprecise and derived from low quality reviews. One literature review (low quality) (53) provided estimates for suicide-related outcomes. All estimates were significant for suicide ideation and ranged from 1.3–1.9, but the CI were large except for the job strain model factors. The association was borderline significant between low control and suicide (RR=1.23, 95% CI 1.00–1.51). Two reviews (moderate or high quality) explored psychotropic medication use (54, 61), and most of the associations were not significant except for psychological demands, job insecurity, and work–life imbalance (RR ranging from 1.1–1.4).

**Figure 9:**
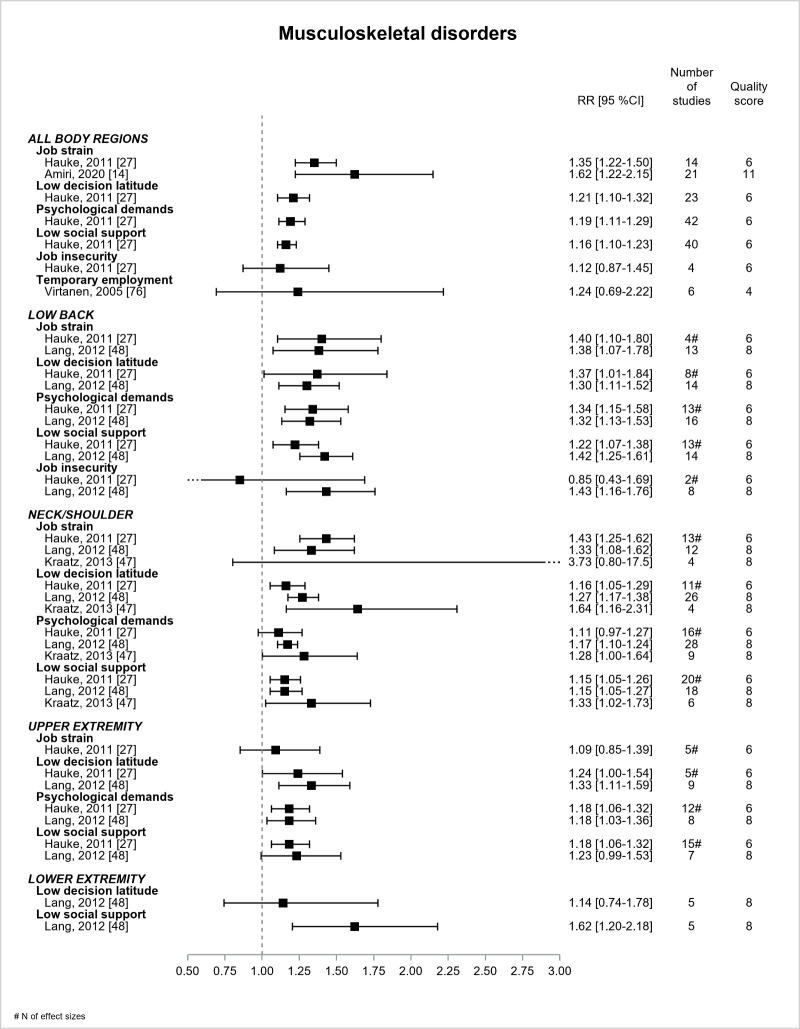
Pooled estimates for the associations between psychosocial work exposures and musculoskeletal disorders. # N of effect sizes.

Figure 9 shows the results for MSD according to the region of pain. There were 5 reviews (14, 27, 47, 48, 76), of low or moderate quality, and all were based on prospective design, except one (76). Job strain model exposures were associated with MSD (all regions), and the two RR for job strain ranged from 1.35 (low quality) to 1.62 (moderate quality). Job strain model exposures were associated with low back pain, the estimates being consistent between the 2 reviews involved (27, 48). Job strain displayed RR ranging between 1.38–1.40. Job insecurity was significantly associated with low back pain in the most recent review (RR=1.43, 95% CI 1.16–1.76) (48). According to three reviews (27, 47, 48), all estimates were significant for the associations between job strain model exposures and neck/shoulder pain, except two estimates. The differences in the estimates between two of these reviews (27, 48) and the third one (47) remained difficult to understand. RR ranging from 1.33–1.43 were found for job strain. According to two reviews (27, 48), low latitude and high demands were associated with upper extremity pain (27, 48). Low support was significant in one review (27) and not in the other (48). There was no significant association between job strain and upper extremity pain. One review (48) found a significant association of low support with lower extremity pain. Contrarily to our expectations, the associations of job strain with MSD were not always more precise over time when two or more reviews were available. The magnitude of the association was found to be approximately consistent between job strain and MSD (all regions), low back and neck/shoulder pain (RR ranged from 1.3–1.6).

**Figure 10:**
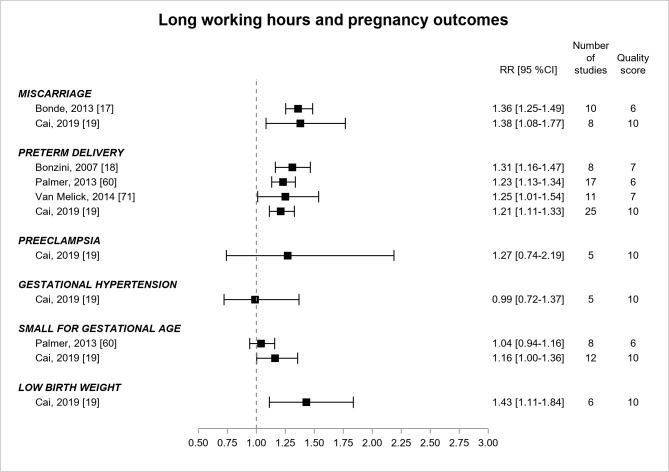
Pooled estimates for the associations between long working hours and pregnancy outcomes.

Pregnancy outcomes (figure 10) were explored in 5 reviews (17–19, 60, 71), with quality increasing from low to moderate over time, that displayed significant associations between long working hours and miscarriage (two estimates of similar magnitude, RR ranging between 1.36–1.38), preterm delivery (3 most recent estimates of similar magnitude, RR ranging between 1.21–1.25), small-for-gestational-age (one borderline significant estimate, RR=1.16, 95% CI 1.00–1.36) (19), and low birth weight (RR=1.43, 95% CI 1.11–1.84) (19).

**Figure 11:**
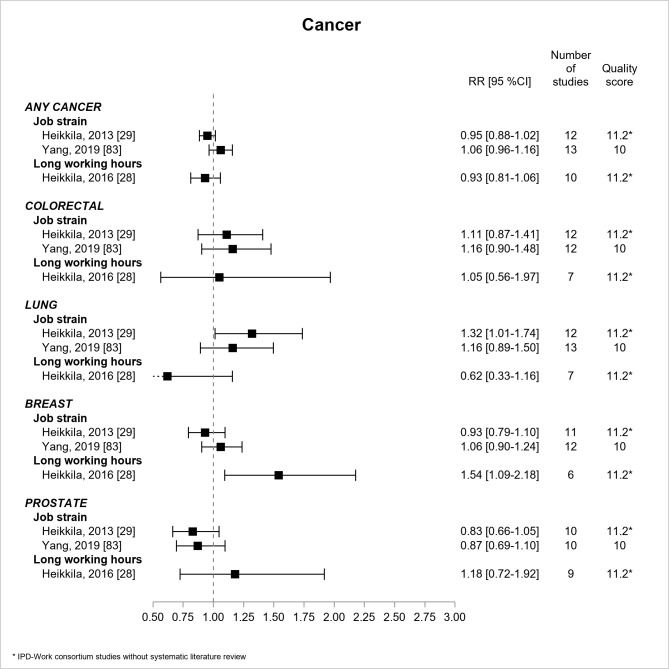
Pooled estimates for the associations between psychosocial work exposures and cancer. * IPD-Work Consortium studies without systematic literature review.

Figure 11 shows the results for cancer. Two IPD-Work consortium studies without literature review (28, 29) and one review (83) explored cancer, and all three were based on prospective design and were of moderate quality. All estimates were non-significant, except two associations, significant though imprecise, between job strain and lung cancer (RR=1.32, 95% CI 1.01–1.74) (29), and between long working hours and breast cancer (RR=1.54, 95% CI 1.09–2.18) (28).

Finally, an IPD-Work consortium study without literature review (33), of moderate quality, did not show an association between job strain and Crohn’s disease and ulcerative colitis.

## Discussion

### Summary of the results

This meta-review of 72 reviews showed that the associations were mainly significant between psychosocial work exposures and cardiovascular diseases (CHD and stroke) and mental disorders, particularly depression, based on the highest quality reviews. The magnitude of the associations was a little stronger for mental disorders than for cardiovascular diseases. High-quality reviews reported significant pooled estimates for job/high strain and long working hours in association with the 3 outcomes of CHD, (ischemic) stroke, and depression, as well as for effort-reward imbalance with CHD, and job insecurity with depression. Based on high-quality reviews, a few other significant associations were found between job strain, job insecurity and diabetes, long working hours and obesity, high strain and physical inactivity, and job insecurity and anxiety and psychotropic medication use. Nevertheless, the consistency over time of the associations varied according to the studied exposure–outcome association.

### Comparison with the literature

There were four previous meta-reviews (7–10), two on the outcomes of cardiovascular diseases (7) and common mental health problems (8), and two others on the exposures of long working hours (10) and workplace bullying (9). Our results are in line with the findings of these meta-reviews. Fishta et al. (7) reported moderate evidence for the associations between psychosocial work factors (mainly job strain) and cardiovascular outcomes. According to Harvey et al (8), there was moderate evidence for the associations of high job demands, low job control, effort–reward imbalance, low justice, role stress, bullying, and low social support with common mental health problems. Nielsen et al (9) showed that bullying was associated with a large number of health outcomes. However, these meta-reviews did not present and compare the results in terms of pooled estimates, and significance, magnitude, precision, and consistency of the associations. Furthermore, they provided narrative information about the associations with cardiovascular diseases and mental health problems, as broad outcomes, but stated no conclusion about specific outcomes. The exception is the meta-review by Rivera et al focusing on long working hours (10) that used literature reviews with meta-analysis and concluded that stroke was the only outcome with moderate evidence in association with this exposure. Thus, our meta-review underlined that long working hours may have an impact on other health outcomes principally CHD and, to a lesser extent, obesity and depression.

### Strengths and limitations of the study

This meta-review had a number of limitations. As a meta-review relies on both the available literature reviews and the primary studies included in each review, it reflects the limitations, including heterogeneity of methods and measurements and sources of bias, of both the included reviews and the primary studies upon which the results of these reviews were based.

This meta-review collected pooled estimates from published literature reviews with meta-analysis. Consequently, the results from reviews without meta-analysis or from published primary studies not included in the reviews were ignored. This approach was probably appropriate for health outcomes such as cardiovascular diseases or mental disorders for which research has accumulated over decades, but may not be for other health outcomes for which reviews with meta-analysis may be missing. As an example, there was one review without meta-analysis for cognitive disorders (86), suggesting some rare uncovered areas in our meta-review. We excluded reviews on all-cause mortality because this outcome was not related to a specific disease or disorder. One systematic literature review with meta-analysis was published on all-cause mortality (68) and was included in our meta-review for CHD mortality. This review showed that low job control only was associated with all-cause mortality. Our meta-review included reviews published until 28 September 2020. Reviews published afterwards include a review by Li et al (87) reporting an association between job strain and diabetes, especially among women, and a review by Mikkelsen et al (88) assessing the evidence for causality whose authors concluded that any of the studied psychosocial work factors was “either likely or unlikely to cause depressive episodes”. Publication bias resulting from the non-publication of primary studies or literature reviews with non-significant results may have overestimated the pooled estimates. Publication bias was noted by the authors of the IPD-Work consortium in one study (43). In our meta-review, 58% of the reviews that explored publication bias did not find this bias whereas 42% found this bias.

For a given exposure–outcome association, there may have been overlap in primary studies included in several reviews, preventing us from pooling all the available estimates, but as we had an interest in examining the pooled estimates over time, this issue may not be a problem. Indeed, our meta-review showed that some recent reviews were able to provide more precise pooled estimates than previous ones, which was consistent with a higher statistical power related to a higher number of primary studies. However, some inconsistencies between reviews were also observed in both magnitude and precision of the association over time and might be explained by differences in the choice of selection criteria. For example, the reviews that included all study designs were more likely to provide higher pooled estimates than those based on prospective design alone. In addition, in case of more than one review for a given association, this allowed us to identify the best quality review (which was not always the most recently published review).

It was not always easy to conclude on the comparison of the magnitude of the associations in order to identify the exposures with the highest magnitude of association, as estimates with higher magnitude were also often those with the highest level of imprecision (largest CI).

There may have been heterogeneity and differences in the measurement of exposure and outcome between primary studies and between reviews. Exposure may differ in terms of assessment method, questionnaire, definition, computation, and cut-off scores utilized. Outcomes may also differ in terms of assessment method, which may lead to outcomes different in nature (for example, depression-related outcomes were very different, as they covered a broad continuum from symptoms to clinical disorder). This heterogeneity was not always taken into account in the included reviews. There was also a major lack of information about exposure duration or cumulative exposure, as most primary studies examined exposure measured at one point in time only, leading to potential misclassification and bias towards the null hypothesis in prospective studies. By contrast, reporting bias related to the measurement of both exposure and outcome (especially for the study of mental health outcomes) may have overestimated the associations. The adjustment variables retained in our review were gender, age, and SES, and if not available the closest minimally adjustment possible. This choice was made to make the results as homogeneous and comparable as possible. In addition, including more adjustment variables may not be appropriate as some variables may be mediators in the studied associations and lead to over adjustment. However, there was no major difference in the results between the retained adjustment and additional adjustment when it was available, though not systematically tested in the reviews. Nevertheless, residual confounding bias may still be possible, as all the primary studies were observational.

Our meta-review included strengths that also deserve to be mentioned.

It was based on a well-defined meta-review protocol, following PRISMA guidelines. We searched in various databases and made a check into the reference lists of the included reviews.

We focused on reviews with meta-analysis to be able to provide pooled estimates and draw more solid and quantitative conclusions. We were thus able to provide information on the significance, magnitude, precision, and consistency over time of the pooled estimates. Furthermore, we were also able to present and compare the results according to specific exposures and outcomes. This strategy was in accordance with the previous meta-review by Rivera et al (10) on long working hours in which literature reviews with meta-analysis were included and specific outcomes were studied, and expanded the knowledge provided by the three other meta-reviews that studied very broad outcomes only (7–9). In addition, we summarized the results for all psychosocial work factors and all health outcomes, which has never been done before. The quality of reviews was assessed in our meta-review using various critical points, including methodological criteria.

We extracted a large amount of information from the included reviews. We collected information about gender differences and other subgroup differences. When heterogeneity was studied, moderate or high level of heterogeneity was found in more than half of the reviews. When subgroup differences were tested, there were in general no or few differences between subgroups, which may suggest that differences in study population may not be a major source of heterogeneity. Only few reviews tested differences according to study characteristics and found few differences. However, our meta-review suggested differences related to study design.

### Perspectives

Methodological issues, which may appear in some primary studies but may not be translated in reviews to date, may be underlined for future research. The assessment of psychosocial work factors should be enlarged to under- or less studied factors, as the literature tends to focus on a limited number of factors (especially job strain). The assessment of psychosocial work factors relied generally on questionnaires (also called subjective assessment methods), and the use of validated scales or questionnaires when available and respect for use recommendations are highly recommended. The use of alternative objective assessment methods remains rare (job-exposure matrix, expert assessment, etc.) and should be expanded in order to compare these methods and provide information on reporting bias. Theorell and Hasselhorn (89) underlined the importance that the exposure–outcome association be “repeatedly being confirmed” whatever the assessment method used (in accordance with the consistency criterion (12)). More information is needed on exposure duration and dose-response associations, as well as time lags and reversibility of the effects. The use of diagnostic instruments/methods for outcome measurement especially for mental health outcomes, but also for other health outcomes, should be more extensive. The study of other health outcomes, that are not cardiovascular and mental health outcomes, should be strengthened. In sum, to further advance the knowledge in this field, high-quality studies are needed and should prioritize: prospective design, study of a larger spectrum of psychosocial work factors, use of objective assessment methods for both exposure and outcome in combination with subjective methods, and study of repeated measures of exposure over time, duration of exposure, dose–response associations, and multiple exposures. More systematic formal testing of subgroup differences in the exposure–outcome associations is suitable, especially between gender, age and SES groups. The impact of study characteristics and methods should also be deepened.

More information is also needed on the mechanisms that may explain the exposure–outcome associations. Firstly, the mechanisms by which psychosocial work factors may impact health outcomes remain poorly understood. For example, robust associations were found for cardiovascular diseases, but the associations for cardiovascular risk factors, including behavioral risk factors, were not as robust as expected. Some rare reviews (90–94) were published on physiological indicators, including immune markers, neuroendocrine stress responses, etc., that may contribute to underlying mechanisms. However, the literature remains sparse and inconclusive for psychosocial work exposures. Secondly, there may be strong overlaps between psychosocial work factors, and more research is suitable on the interrelations between these factors, and the underlying causal mechanisms. Harvey et al (8) attempted to construct a more unified model for psychosocial work factors and suggested overlapping concepts. There may also be a need for more research on the determinants of psychosocial work factors, such as economic, social, and political factors at macro-level and company-level organizational factors. More studies and reviews exploring macro- or company-level factors and psychosocial work factors in association with health outcomes may be informative to better understand the interrelations between these factors and elaborate preventive prospects.

Finally, our meta-review was not designed to capture reviews of intervention studies focusing on psychosocial or organizational intervention at the workplace. Such reviews remain very rare (95), as well as high-quality intervention studies themselves, but would be particularly useful to provide information on the effectiveness of such intervention on well-defined health outcomes, and to make progress towards causality.

### Concluding remarks

Increasing our understanding of the effects of psychosocial work factors on health outcomes is crucial. To this end, it is worth taking advantage of the existing literature, which may not be used fully to date. Given the large corpus of the literature available on the topic of psychosocial work factors and health outcomes, more high-quality literature reviews providing pooled estimates from meta-analysis are an asset. Updates of these reviews with meta-analysis are also needed in order to provide up-to-date pooled estimates. This information may be of upmost importance to define preventive strategies oriented towards the psychosocial work environment. Finally, our present meta-review may also be seen as a reflection of all the results that are still missing and waiting for more research.

### Conflict of interest

The authors declare no conflicts of interest.

### Funding

This project was supported by the French National Research Program for Environmental and Occupational Health of ANSES, France (grant number: EST-2016/1/49) and by ETUI, Brussels, Belgium (grant number: 1851-320)

## Supplementary material

Supplementary material
