# Supplementary material for: Psychosocial work exposures and health outcomes: a meta-review of 72 literature reviews with meta-analysis
Source: Scand J Work Environ Health. 2021 Sep 30;47(7):489–508. doi: 10.5271/sjweh.3968 (PMC8504166; doi:10.5271/sjweh.3968)
Supplement: Supplementary material [file SJWEH-47-489-S001.pdf]

Psychosocial work exposures and health outcomes: a meta-review of literature reviews with meta-analysis[1]

by Isabelle Niedhammer, PhD,[2] Sandrine Bertrais, PhD, Katrina Witt, DPhil

[1] Supplementary material

[2] Correspondence to: Isabelle Niedhammer, INSERM, Univ Angers, Univ Rennes, EHESP, Irset (Institut de recherche en santé, environnement et travail) - UMR_S 1085, ESTER Team, Angers, France [E-mail: isabelle.niedhammer@inserm.fr]

Appendix 1. List of keywords used in the search strategy

Appendix 2. Quality assessment of literature reviews and IPD-Work consortium studies

Supplementary Table S1 (part 1). Description of the included reviews

Supplementary Table S1 (part 2). Description of the included reviews

Supplementary Table S2. Quality assessment of the included reviews

Supplementary Table S3. Full-text articles excluded and reason for exclusion

Appendix 1. List of keywords used in the search strategy

			**Keywords**
		Meta-analysis	Meta-analysis, metaanalysis, meta-analyses, metaanalyses, meta-analytic, metaanalytic, pooled
AND		Work environment	Work*, occupation*, job, employ*
AND		All psychosocial work factors	Psychosocial, stress*
	OR	Job strain model	Strain, demand*, control, latitude, support, influence, development, discretion, authority, freedom, relation*
	OR	Long working hours	Hour*, time, overtime, overwork
	OR	Effort-reward imbalance model	Effort, reward*, imbalance, esteem, respect, promotion
	OR	Job insecurity, temporary employment	Secur*, insecur*, precar*, temporary
	OR	Workplace bullying	Bullying, harassment, violence*, conflict*, mobbing
	OR	Organizational injustice	Injustice, justice
	OR	Quality of leadership	Leadership
	OR	Work-family conflict	Conflict*, imbalance
	OR	Role stressors	Role
	OR	Predictability	Predictability

All keywords were used for search in the titles and keywords related to meta-analysis were also used for search in the abstracts

Appendix 2. Quality assessment of literature reviews and IPD-Work consortium studies

8 critical points

1) systematic literature review (without/with guidelines)

2) study design (prospective design mainly, prospective design only)

3) adjustment (close adjustment, expected adjustment)

4) quality assessment of primary studies and/or evidence (one assessment only, both)

5) appropriate statistical methods for meta-analysis (fixed effects model without checked conditions, random effects model)

6) subgroup and/or sensitivity analysis (gender analysis in studies among both genders, analysis in addition to gender analysis)

7) heterogeneity (study of, low heterogeneity i.e. <50%)

8) publication bias (study of, no publication bias)

○ low quality

◘ moderate quality

● high quality

○◘● or 0-1-2 for each point/criterion

∅ not applicable (for criteria 1, 4, and 8 in the case of IPD-Work consortium studies without literature review)

Overall score 0 (lowest score)-16 (highest score)

A weighted score was calculated for IPD-Work consortium studies without literature review (weight of 8/5)

Low scores: 0-7

Moderate scores: 8-11

High scores: 12-16

Nota Bene: IPD-Work consortium studies without literature review were underlined in yellow in Supplementary Table S1 and a * was added to their quality assessment to indicate that only 5 criteria among 8 were rated.

Supplementary Table S1 (part 1). Description of the included reviews

	Publication date	Ref number	Review / guidelines	Design(s) of primary studies	Other inclusion criteria	Number of primary studies	Outcome(s)	Exposure(s)	Main results: Pooled estimate(s) (95% CI)
Amiri	2020	14	Literature review PRISMA	Longitudinal	Before Jan. 2019	21	Musculoskeletal pain	Job strain (21 studies)	1.62 (1.22-2.15), heterogeneity: I^2=91.6%
Aronsson<br><br>Not in the extraction	2017	15	Literature review PRISMA	Prospective, case control, randomized intervention	1990-Aug. 2013<br>Western world (Europe, North America, Australia and New Zealand)<br>n>100<br>Results controlled for at least age and gender	25	Burnout (emotional exhaustion)	 Low control (9 studies)<br>Job demands (11 studies)<br>Low support (7 studies)<br>Injustice (3 studies)<br>Low reward (2 studies)<br>Job insecurity (3 studies)<br>Emotional demands (5 studies)	Adjusted for at least age and gender :<br>1.63 (1.53-1.75)<br>2.53 (2.36-2.71)<br>1.81 (1.68-1.95)<br>2.86 (2.22-3.70)<br>1.86 (1.37-2.52)<br>1.39 (1.22-1.57)<br>2.95 (2.40-3.62)
Babu	2014	16	Literature review PRISMA	Cohort, case-control	1908-Jan. 2012	9	Hypertension (blood pressure >140/90 mmHg)	Job strain (9 studies)	1.29 (1.14-1.47), heterogeneity: I^2=78%
Bonde	2013	17	Literature review	Prospective cohort, retrospective cohort, case-control, cross-sectional	1966-June 2012<br>Peer-reviewed studies<br>English language	10	Miscarriage	Long working hours ≥40–52 hours a week (10 studies)	1.36 (1.25–1.49), heterogeneity: Q=22.2, p=0.020
Bonzini	2007	18	Literature review	Cohort, case-control, cross-sectional	1966-Dec. 2005<br>Abstract in English	8	Preterm delivery	Long working hours ≥40 hours a week (8 studies)	1.31 (1.16-1.47), heterogeneity: Q=4.33, p=0.74
Cai	2019	19	Literature review PRISMA	All designs (except case studies and reviews)	Before March 2019	59	Pregnancy outcomes	Long working hours ≥40 hours a week	--- Preterm delivery (25 studies):<br>1.21 (1.11-1.33), heterogeneity: I^2=30%<br>--- Miscarriage (8 studies):<br>1.38 (1.08-1.77), heterogeneity: I^2=73%<br>--- Preeclampsia (5 studies):<br>1.27 (0.74-2.19), heterogeneity: I^2=84%<br>--- Gestational hypertension (5 studies):<br>0.99 (0.72-1.37), heterogeneity: I^2=62%

	Publication date	Ref number	Review / guidelines	Design(s) of primary studies	Other inclusion criteria	Number of primary studies	Outcome(s)	Exposure(s)	Main results:<br>Pooled estimate(s) (95% CI)
									--- Small for gestational age (12 studies):<br>1.16 (1.00-1.36), heterogeneity: I^2=57%<br>--- Low birth weight (6 studies):<br>1.43 (1.11-1.84), heterogeneity: I^2=0%
Cosgrove	2012	20	Literature review<br>MOOSE	Cohort, case-control, cross-sectional	Up to March 2010<br>English and non-English language	9	Type 2 diabetes	 Job demands (4 studies)<br><br>Low decision latitude (4 studies)<br><br>Job strain (4 studies)<br><br>Low social support (4 studies)<br><br>Long working hours (3 studies)	Most adjusted models:<br>0.95 (0.81-1.09), heterogeneity: I^2=0%, Cochran's Q-test p=NS<br>1.04 (0.86-1.21), heterogeneity: I^2=12%, Cochran's Q-test p=NS<br>1.08 (0.84-1.32), heterogeneity: I^2=39%, Cochran's Q-test p=NS<br>1.04 (0.88-1.20), heterogeneity: I^2=0%, Cochran's Q-test p=NS<br>0.81 (-0.10-1.71), heterogeneity: I^2=67%, Cochran's Q-test $p<0.05$
Descatha	2020	21	Literature review<br>Navigation Guide<br>PRISMA	Cohort, case-control	Up to May 2018, update in April 2020<br>English language	22	Stroke (incidence, mortality)	Long working hours (reference group: 35-40 h/week)	Adjusted for at least age, sex, and SES:<br>Working hours 41-48 h/week:<br>--- incidence (18 cohort studies):<br>1.04 (0.94-1.14), heterogeneity: I^2=0%<br>--- mortality (12 cohort studies):<br>1.01 (0.91-1.12), heterogeneity: I^2=0%<br>Working hours 49-54 h/week:<br>--- incidence (17 cohort studies):<br>1.13 (1.00-1.28), heterogeneity: I^2=0%<br>--- mortality (11 cohort studies):<br>1.13 (0.99-1.29), heterogeneity: I^2=0%<br>Working hours ≥ 55 h/week:<br>--- incidence (7 cohort studies):<br>1.35 (1.13-1.61), heterogeneity: I^2=3%<br>--- mortality (10 cohort studies):<br>1.08 (0.89-1.31), heterogeneity: I^2=20%
Dragano	2017	22	IPD-Work Consortium	Cohort		11	Coronary heart disease	Effort-reward imbalance (ERI)	Adjusted for age, sex and SES:<br>1.19 (1.04-1.38)
Duchaine	2020	23	Literature review<br>PRISMA<br>MOOSE	Prospective	1946-Oct. 2017, update in Feb. 2019	13	Sickness absence due to a diagnosed mental disorder	 <br>Job strain (8 studies)<br>Psychological demands (7 studies)<br>Low job control (5 studies)<br>Low social support (9 studies)<br>Effort-reward imbalance (3	Adjusted for age, sex, and SES or most of them:<br>1.47 (1.24-1.74), heterogeneity: I^2=58%<br>1.23 (1.04-1.45), heterogeneity: I^2=56%<br><br>1.25 (1.02-1.53), heterogeneity: I^2=72%<br>1.12 (0.99-1.26), heterogeneity: I^2=45%<br>1.66 (1.37-2.00), heterogeneity: I^2=34%

	Publication date	Ref number	Review / guidelines	Design(s) of primary studies	Other inclusion criteria	Number of primary studies	Outcome(s)	Exposure(s)	Main results: Pooled estimate(s) (95% CI)
								studies)<br>Low reward (3 studies)	 1.76 (1.49-2.08), heterogeneity: I^2=0%
Ferrie	2016	24	IPD-Work Consortium	Prospective cohort		19	Diabetes	Job insecurity	Adjusted for age, sex and SES (15 studies):<br>1.15 (1.04–1.28)
Fransson	2012	25	IPD-Work Consortium	Cross-sectional and longitudinal analyses based on prospective cohorts		14	Leisure-time physical inactivity	High strain, passive and active job (reference group: low strain)	Cross-sectional associations adjusted for sex and age (14 studies):<br>--- high strain 1.36 (1.25-1.48)<br>--- passive job 1.34 (1.23-1.47)<br>--- active job 1.00 (0.93-1.06)<br>Prospective associations adjusted for sex, age, SES and smoking (6 studies):<br>--- high strain 1.21 (1.11-1.32)<br>--- passive job 1.20 (1.11-1.30)<br>--- active job 1.07 (0.99-1.15)
Fransson	2015	26	IPD-Work Consortium	Prospective cohort		14	Stroke (overall, ischemic, hemorrhagic)	Job strain	Age- and sex-adjusted results:<br>--- overall stroke (14 studies):<br>1.09 (0.94-1.26), heterogeneity: I^2=21.6%<br>Age-, sex- and SES-adjusted results:<br>--- ischemic stroke (13 studies):<br>1.18 (1.00-1.39)<br>--- hemorrhagic stroke (12 studies):<br>0.95 (0.72-1.27)
Hauke	2011	27	Literature review	Longitudinal	2000-2009<br>English language	47	Musculoskeletal disorders (all body regions)<br>Neck/shoulder<br>Upper extremities<br>Low back	Low social support<br>High job demands<br>Low job control<br>High job strain<br>High job insecurity	The most adjusted models:<br>All body regions:<br>- Low social support (40 studies, 51 estimates): 1.16 (1.10-1.23)<br>- High job demands (42 studies, 43 estimates): 1.19 (1.11-1.29)<br>- Low job control (23 studies, 26 estimates): 1.21 (1.10-1.32)<br>- High job strain (14 studies, 22 estimates): 1.35 (1.22-1.50)<br>- High job insecurity (4 studies, 4 estimates): 1.12 (0.87-1.45)<br><br>Low back:<br>- Low social support (13 estimates):<br>1.22 (1.07-1.38)<br>- High job demands (13 estimates):<br>1.34 (1.15-1.58)

	Publication date	Ref number	Review / guidelines	Design(s) of primary studies	Other inclusion criteria	Number of primary studies	Outcome(s)	Exposure(s)	Main results: Pooled estimate(s) (95% CI)
									- Low job control (8 estimates): 1.37 (1.01-1.84)<br>- High job strain (4 estimates): 1.40 (1.10-1.80)<br>- High job insecurity (2 estimates): 0.85 (0.43-1.69)<br><br>Neck/shoulder:<br>- Low social support (20 estimates): 1.15 (1.05-1.26)<br>- High job demands (16 estimates): 1.11 (0.97-1.27)<br>- Low job control (11 estimates): 1.16 (1.05-1.29)<br>- High job strain (13 estimates): 1.43 (1.25-1.62)<br><br>Upper extremities:<br>- Low social support (15 estimates): 1.18 (1.06-1.32)<br>- High job demands (12 estimates): 1.18 (1.06-1.32)<br>- Low job control (5 estimates): 1.24 (1.00-1.54)<br>- High job strain (5 estimates): 1.09 (0.85-1.39)
Heikkila<br><br>Not in the extraction	2016	28	IPD-Work Consortium	Prospective cohort		14	Cancer (any cancer, colorectal, lung, breast, prostate)	Long working hours (reference group: 35-40 h/week)	Age- and sex- adjusted results:<br>Working hours 41-48 h/week:<br>--- any cancer (12 studies):<br>0.97 (0.88-1.06), heterogeneity: I^2=2.4%<br>--- colorectal cancer (10 studies):<br>1.01 (0.76-1.36), heterogeneity: I^2=0%<br>--- lung cancer (10 studies):<br>0.84 (0.57-1.24), heterogeneity: I^2=0%<br>--- breast cancer (10 studies):<br>0.96 (0.73-1.27), heterogeneity: I^2=19.1%<br>--- prostate cancer (9 studies):<br>0.95 (0.76-1.19), heterogeneity: I^2=0%<br>Working hours 49-54 h/week:<br>--- any cancer (11 studies):

	Publication date	Ref number	Review / guidelines	Design(s) of primary studies	Other inclusion criteria	Number of primary studies	Outcome(s)	Exposure(s)	Main results: Pooled estimate(s) (95% CI)
									1.09 (0.97-1.23), heterogeneity: I^2=0%<br>--- colorectal cancer (8 studies):<br>1.59 (0.96-2.61), heterogeneity: I^2=27.5%<br>--- lung cancer (5 studies):<br>0.70 (0.35-1.41), heterogeneity: I^2=0%<br>--- breast cancer (9 studies):<br>0.99 (0.62-1.57), heterogeneity: I^2=15.4%<br>--- prostate cancer (10 studies):<br>1.54 (1.07-2.22), heterogeneity: I^2=29.0%<br>Working hours ≥ 55 h/week:<br>--- any cancer (10 studies):<br>0.93 (0.81-1.06), heterogeneity: I^2=0%<br>--- colorectal cancer (7 studies):<br>1.05 (0.56-1.97), heterogeneity: I^2=37.9%<br>--- lung cancer (7 studies):<br>0.62 (0.33-1.16), heterogeneity: I^2=0%<br>--- breast cancer (6 studies):<br>1.54 (1.09-2.18), heterogeneity: I^2=0%<br>--- prostate cancer (9 studies):<br>1.18 (0.72-1.92), heterogeneity: I^2=35.9%
Heikkila	2013	29	IPD-Work Consortium	Prospective cohort		12	Cancer (any cancer, colorectal, lung, breast, prostate)	Job strain	Age- and sex- adjusted results:<br>--- any cancer (12 studies):<br>0.95 (0.88-1.02), heterogeneity: I^2=0%<br>--- colorectal cancer (12 studies):<br>1.11 (0.87-1.41), heterogeneity: I^2=0%<br>--- lung cancer (12 studies):<br>1.32 (1.01-1.74), heterogeneity: I^2=0%<br>--- breast cancer (11 studies):<br>0.93 (0.79-1.10), heterogeneity: I^2=0%<br>--- prostate cancer (10 studies):<br>0.83 (0.66-1.05), heterogeneity: I^2=0%
Heikkila	2020	30	IPD-Work Consortium	Prospective cohort		11	Hospital record of peripheral artery disease	Job strain	Age- and sex-adjusted:<br>1.46 (1.17-1.83), heterogeneity: I^2=21.4%
Heikkila	2012	31	IPD-Work Consortium	Cross-sectional and		15	Tobacco smoking	Job strain	Meta-analysis of cross-sectional associations adjusted for sex, age and

	Publication date	Ref number	Review / guidelines	Design(s) of primary studies	Other inclusion criteria	Number of primary studies	Outcome(s)	Exposure(s)	Main results: Pooled estimate(s) (95% CI)
				prospective analyses of prospective cohorts					SES (15 studies), with job strain as outcome:<br>--- non-smokers: reference category<br>--- ex smokers: 1.00 (0.93-1.06), heterogeneity: I^2=69.3%<br>--- current smokers: 1.11 (1.03-1.18), heterogeneity: I^2=67.8%<br>Prospective associations based on the pooled individual-level data, adjusted for sex, age and SES (6 studies), OR (95% CI) for being smoker at follow-up among baseline never- and ex-smokers:<br>--- job strain at baseline: 1.03 (0.87-1.21)
Heikkila	2012	32	IPD-Work Consortium	Cross-sectional and prospective analyses of prospective cohorts		12	Alcohol intake	Job strain	Meta-analysis of cross-sectional associations adjusted for sex, age and SES (12 studies), with job strain as outcome:<br>--- non-drinkers:<br>1.10 (1.05-1.14), heterogeneity: I^2=0.0%<br>--- moderate drinkers: reference category<br>--- intermediate drinkers:<br>0.92 (0.86-0.99), heterogeneity: I^2=0.0%<br>--- heavy drinkers:<br>1.12 (1.00-1.26), heterogeneity: I^2=61.9%<br>Prospective associations based on the pooled individual-level data, adjusted for sex, age and SES (4 studies), OR (95% CI) for becoming intermediate or heavy drinker among baseline moderate and non-drinkers:<br>--- job strain at baseline: 0.90 (0.79-1.01)
Heikkila	2014	33	IPD-Work Consortium	Prospective cohort		11	Inflammatory bowel diseases (Crohn's disease, ulcerative colitis)	Job strain	Age- and sex-adjusted:<br>--- Crohn's disease: 0.89 (0.52-1.52), heterogeneity: I^2=0.0%<br>--- Ulcerative colitis: 1.14 (0.80-1.61), heterogeneity: I^2=27.1%
Huang	2015	34	Literature review MOOSE	Prospective cohort	Up to July 2014	6	Stroke (overall, ischemic, hemorrhagic)	High strain, passive and active job (reference group: low strain)	All stroke (6 studies):<br>--- high strain: 1.22 (1.01-1.47), heterogeneity: I^2=9%<br>--- passive job: 1.07 (0.90–1.28),

	Publication date	Ref number	Review / guidelines	Design(s) of primary studies	Other inclusion criteria	Number of primary studies	Outcome(s)	Exposure(s)	Main results: Pooled estimate(s) (95% CI)
									heterogeneity: I^2=41%<br>--- active job: 1.01 (0.86-1.18), heterogeneity: I^2=17%<br>Ischemic stroke (3 studies):<br>--- high strain: 1.58 (1.12-2.23), heterogeneity: I^2=90%<br>Hemorrhagic stroke (2 studies):<br>--- high strain: 1.37 (0.73-2.58), heterogeneity: I^2=9%
Kang	2012	35	Literature review	Cohort, case-control	Up to Sept. 2011<br>English language	11	Cardiovascular disease	Long working hours ≥45–55 hours a week	1.37 (1.11-1.70), heterogeneity: p=0.037
Kim	2016	36	Literature review MOOSE	Prospective	Jan. 2005- Dec. 2014	6	Depressive symptoms	Job insecurity (6 studies)	1.29 (1.06–1.57), heterogeneity: I^2=89%
Kivimaki	2013	37	IPD-Work Consortium	Prospective cohort		7	Coronary heart disease	Job strain	Adjusted for age, sex, and cohort: 1.25 (1.06–1.47)
Kivimaki<br><br>Update of Kivimaki E 2014	2015	38	Literature review	Prospective cohort		27	Coronary heart disease	Job strain	1.33 (1.19-1.49)
Kivimaki<br><br>Idem Steptoe ARPH 2013	2014	39	Literature review	Prospective cohort		26	Coronary heart disease	Job strain	1.34 (1.18-1.51), heterogeneity: I^2=21%
Kivimaki<br><br>Not in the extraction	2018	40	IPD-Work Consortium	Prospective cohort		8	Venous thromboembolism (deep-vein thrombosis, pulmonary embolism)	Long working hours ≥55 hours a week (reference group: 35-40 h/week)	Adjusted for age, sex, cohort, and SES:<br>--- Venous thromboembolism: 1.5 (1.1-2.1)<br>--- Deep-vein thrombosis: 1.7 (1.1-2.5)<br>--- Pulmonary embolism: 1.4 (0.8-2.4)
Kivimaki	2017	41	IPD-Work Consortium	Prospective cohort		8	Atrial fibrillation	Long working hours	Adjusted for age, sex, and SES: 1.42 (1.13-1.80), heterogeneity: I^2=0%
Kivimaki	2015	42	Literature review PRISMA	Cohort	Up to Aug 2014	8	Weight gain<br>Obesity	Job strain	Adjusted for age, sex and SES:<br>Weight gain (4 studies):<br>--- random-effect meta-analysis: 1.12 (0.99-1.28), heterogeneity: I^2=48.4%

	Publication date	Ref number	Review / guidelines	Design(s) of primary studies	Other inclusion criteria	Number of primary studies	Outcome(s)	Exposure(s)	Main results: Pooled estimate(s) (95% CI)
									Obesity (4 studies):<br>--- random-effect meta-analysis:<br>1.01 (0.88-1.16), heterogeneity: I^2=18.3%
Kivimaki	2012	43	IPD-Work Consortium	Prospective cohort		13	Coronary heart disease (fatal and non-fatal events)	Job strain	Adjusted for age, sex and SES (13 studies):<br>1.17 (1.05-1.31)
Kivimaki	2015	44	Literature review<br>PRISMA<br>IPD-Work Consortium	Prospective cohort	Up to Aug 2014<br>English language	22 (CHD)<br>14 (Stroke)	Coronary heart disease and stroke	Long working hours ≥55 hours a week (reference group: 35-40 h/week)	Adjusted for age, sex, and SES:<br>--- coronary heart disease:<br>1.13 (1.02–1.26), heterogeneity: I^2=0%<br>--- stroke:<br>1.33 (1.11–1.61), heterogeneity: I^2=0%
Kivimaki	2015	45	Literature review<br>PRISMA<br>IPD-Work Consortium	Prospective cohort	Up to April 2014<br>English language	23	Type 2 diabetes	Long working hours ≥55 hours a week (reference group: 35-40 h/week)	Adjusted for age and sex:<br>1.07 (0.89–1.27), heterogeneity: I^2=53%
Kivimaki	2006	46	Literature review	Prospective cohort	Up to Jan. 2006	14	Coronary heart disease or cardiovascular mortality	Job strain<br>Effort-reward imbalance<br>Organizational injustice	Age- and gender-adjusted:<br>--- job strain (10 studies):<br>1.45 (1.15–1.84), heterogeneity: Q=17.5, p=0.04<br>--- effort-reward imbalance (4 studies):<br>1.58 (0.84–2.97), heterogeneity: Q=3.9, p<0.05<br>--- organizational injustice (2 studies):<br>1.62 (1.24–2.13), heterogeneity: not tested
Kraatz	2013	47	Literature review<br>PRISMA	Prospective longitudinal	Up to May 2009<br>English or German language	18	Neck and/or shoulder complaints or disorders (exclusion of injuries or malign diseases)	 Job demands (9 studies)<br>Low job control (4 studies)<br>Low social support (6 studies)<br>Job strain (4 studies)	Adjusted for at least one physical factor:<br>1.28 (1.00-1.64), heterogeneity: I^2=77%<br>1.64 (1.16-2.31), heterogeneity: I^2=57%<br>1.33 (1.02-1.73), heterogeneity: I^2=67%<br>3.73 (0.80-17.54), heterogeneity: I^2=84%
Lang	2012	48	Literature review	Longitudinal	Up to Aug. 2009<br>Peer-reviewed articles<br>English or German	50	Musculoskeletal problems	High job demands<br>Low job control<br>High job strain<br>Low social support<br>Low job security	Lower back:<br>High job demands (16 studies):<br>1.32 (1.13-1.53), heterogeneity: I^2=62.1%<br>Low job control (14 studies):

	Publication date	Ref number	Review / guidelines	Design(s) of primary studies	Other inclusion criteria	Number of primary studies	Outcome(s)	Exposure(s)	Main results: Pooled estimate(s) (95% CI)
					language<br>Working populations in industrialized countries				1.30 (1.11-1.52), heterogeneity: I^2=44.7%<br>High job strain (13 studies):<br>1.38 (1.07-1.78), heterogeneity: I^2=49.4%<br>Low social support (14 studies):<br>1.42 (1.25-1.61), heterogeneity: I^2=0.0%<br>Low job security (8 studies):<br>1.43 (1.16-1.76), heterogeneity: I^2=48.9%<br><br>Neck and/or shoulder:<br>High job demands (28 studies):<br>1.17 (1.10-1.24), heterogeneity: I^2=18.6%<br>Low job control (26 studies):<br>1.27 (1.17-1.38), heterogeneity: I^2=4.3%<br>High job strain (12 studies):<br>1.33 (1.08-1.62), heterogeneity: I^2=46.0%<br>Low social support (18 studies):<br>1.15 (1.05-1.27), heterogeneity: I^2=2.3%<br><br>Upper extremity:<br>High job demands (8 studies):<br>1.18 (1.03-1.36), heterogeneity: I^2=0.0%<br>Low job control (9 studies):<br>1.33 (1.11-1.59), heterogeneity: I^2=29.0%<br>Low social support (7 studies):<br>1.23 (0.99-1.53), heterogeneity: I^2=46.6%<br><br>Lower extremity:<br>Low job control (5 studies):<br>1.14 (0.74-1.78), heterogeneity: I^2=58.3%<br>Low social support (5 studies):<br>1.62 (1.20-2.18), heterogeneity: I^2=12.6%
Li	2020	49	Literature review	Cohort studies	Up to Aug. 2019	35	Ischaemic heart disease	Long working hours (reference group: 35-40 h/week)	Adjusted for at least age, sex, and SES:

	Publication date	Ref number	Review / guidelines	Design(s) of primary studies	Other inclusion criteria	Number of primary studies	Outcome(s)	Exposure(s)	Main results: Pooled estimate(s) (95% CI)
			Navigation Guide<br>PRISMA<br>GATHER	(retrospective or prospective), case-control studies			(incidence, mortality)		Working hours 41-48 h/week:<br>--- incidence (20 cohort studies):<br>0.98 (0.91-1.07), heterogeneity: I^2=0%<br>--- mortality (13 cohort studies):<br>0.99 (0.88-1.12), heterogeneity: I^2=8%<br>Working hours 49-54 h/week:<br>--- incidence (18 cohort studies):<br>1.05 (0.94-1.17), heterogeneity: I^2=0%<br>--- mortality (11 cohort studies):<br>1.01 (0.82-1.25), heterogeneity: I^2=13%<br>Working hours ≥ 55 h/week:<br>--- incidence (22 cohort studies):<br>1.13 (1.02-1.26), heterogeneity: I^2=5%<br>--- mortality (16 cohort studies):<br>1.17 (1.05-1.31), heterogeneity: I^2=0%
Linton<br><br>Not in the extraction	2015	50	Literature review<br>PRISMA	Prospective or randomized	1990-Oct.2012<br>English language	22	Sleep disturbances	High demands (6 studies)<br>Low control (6 studies)<br>Job strain (4 studies)<br>Low social support (4 studies)<br>Organizational injustice (3 studies)<br>Effort-reward imbalance (3 studies)<br>Bullying (3 studies)	Least adjusted:<br>1.48 (1.28-1.72)<br>1.08 (0.89-1.28)<br>1.35 (1.17-1.56)<br>1.35 (1.11-1.64)<br>1.25 (1.00-1.56)<br><br>1.44 (0.98-2.11)<br><br>1.42 (0.75-2.68)
Madsen	2017	51	Literature review<br>PRISMA<br>IPD-Work Consortium	Cohort	Up to Sept. 2015<br>Peer-reviewed journals<br>English language	20	Clinical depression (published studies)<br>Hospital-treated clinical depression (unpublished studies)	Job strain	Published studies (6 studies):<br>1.77 (1.47–2.13), heterogeneity: I^2=24.2%<br>Adjusted for age, sex, cohabitation, and SES:<br>Unpublished studies (14 studies):<br>1.22 (1.02–1.47), heterogeneity: I^2=24.8%
Magnavita	2019	52	Literature review<br>PRISMA	Cross-sectional, retrospective, case-control and prospective	Up to April 2019<br>English abstract and keyword, full-text in English or Italian	7	Sleep problems	Workplace violence	2.55 (1.77-3.66), heterogeneity: I^2=96%

	Publication date	Ref number	Review / guidelines	Design(s) of primary studies	Other inclusion criteria	Number of primary studies	Outcome(s)	Exposure(s)	Main results:<br>Pooled estimate(s) (95% CI)
Milner	2018	53	Literature review PRISMA	Prospective cohort, case–control, retrospective mortality, cross-sectional or intervention trial	Up to Jan. 2017 No restrictions on publication status or language	22	Suicide ideation<br><br><br><br><br><br><br><br><br><br><br><br><br>Suicide death	 Low social support (5 studies)<br><br>Low control (6 studies)<br><br>Demands (7 studies)<br><br>Effort-reward imbalance (3 studies)<br>Job insecurity (5 studies)<br><br>Job strain (2 studies)<br><br>Low support (2 studies)<br>Low control (4 studies)<br><br>Demands (4 studies)	Unadjusted:<br>1.45 (1.01-2.08), heterogeneity: I^2=78.0%<br>1.32 (1.14-1.54), heterogeneity: I^2=86.0%<br>1.35 (1.15-1.59), heterogeneity: I^2=88.2%<br>1.81 (1.30-2.52), heterogeneity: I^2=63.3%<br>1.91 (1.22-2.99), heterogeneity: I^2=96.1%<br>1.77 (1.42-2.20), heterogeneity: I^2=0.0%<br><br>1.16 (0.98-1.38), heterogeneity: I^2=8.7%<br>1.23 (1.00-1.51), heterogeneity: I^2=97.9%<br>1.08 (0.84-1.38), heterogeneity: I^2=93.0%
Milner	2019	54	Literature review PRISMA	Prospective cohort, case–control, retrospective mortality, cross-sectional or intervention trial	Up to July 2018	18	Use of psychotropic medications	Low job control:<br>--- OR reported (3 studies)<br><br>--- RR reported (4 studies)<br><br>Job demands:<br>--- OR reported (3 studies)<br><br>--- RR reported (5 studies)<br><br>Job strain:<br>--- OR reported (2 studies)<br><br>Low social support:<br>--- OR reported (2 studies)<br>--- RR reported (4 studies)<br><br>Effort-reward imbalance:<br>--- RR reported (2 studies)<br>Precarity:<br>--- RR reported (2 studies)<br>Work-family conflict:<br>--- RR reported (3 studies)	 1.16 (0.93-1.39), heterogeneity: I^2=39.3%<br>1.02 (0.76-1.27), heterogeneity: I^2=79.3%<br><br>1.39 (1.06-1.71), heterogeneity: I^2=69.0%<br>1.16 (1.02-1.31), heterogeneity: I^2=58.7%<br><br>1.18 (0.95-1.41), heterogeneity: I^2=43.5%<br><br>1.14 (0.91-1.37), heterogeneity: I^2=0.0%<br>0.98 (0.89-1.06), heterogeneity: I^2=56.2%<br><br>1.14 (0.93-1.35), heterogeneity: I^2=0.0%<br><br>0.99 (0.84-1.14), heterogeneity: I^2=0.0%<br><br>1.26 (1.03-1.48), heterogeneity: I^2=0.0%

	Publication date	Ref number	Review / guidelines	Design(s) of primary studies	Other inclusion criteria	Number of primary studies	Outcome(s)	Exposure(s)	Main results:<br>Pooled estimate(s) (95% CI)
								Working hours:<br>--- RR reported (3 studies)	 1.05 (0.93-1.17), heterogeneity: I^2=0.0%
Nielsen	2020	55	Literature review<br>PRISMA<br>MOOSE	Cross-sectional, prospective, retrospective<br>,<br>case–control, experimental designs	Up to Dec. 2017, update in Aug. 2018<br>English, German, French, or Scandinavian languages (Danish, Norwegian, Swedish)	16	Sleep problems	Workplace bullying	Cross-sectional studies (13 studies): 2.31 (1.93-2.75), heterogeneity: I^2=90.1%<br>Prospective studies (5 studies): 1.62 (1.00-2.63), heterogeneity: I^2=90.2%<br>No significant difference (p=0.18) between cross-sectional and prospective results
Nieuwenhuijsen	2010	56	Literature review	Prospective	Up to Oct 2008	7	Absenteeism due to mental health problems or a high level of psychological complaints as reflected in a score above a cut-off point on a validated questionnaire for fatigue, stress or non-specific mental ill-health or an adjustment disorder	Job demands (3 studies)<br>Low job control (2 studies)<br>Low co-worker support (2 studies)<br>Low supervisor support (3 studies)<br>Procedural injustice (2 studies)<br>Relational injustice (2 studies)<br>Effort-reward imbalance (4 studies)	1.35 (1.22–1.50)<br>1.22 (1.10–1.36)<br>1.24 (1.13–1.37)<br><br>1.24 (1.13–1.35)<br><br>1.78 (1.59–2.00)<br>1.51 (1.35–1.69)<br>1.98 (1.78–2.20)
Nyberg	2014	57	IPD-Work Consortium	Cohort		13	Type 2 diabetes	Job strain	Adjusted for age, sex, and SES:<br>1.15 (1.06–1.25), heterogeneity: I^2=0%
Nyberg	2012	58	IPD-Work Consortium	Cross-sectional and longitudinal analyses of prospective cohorts		13	Obesity as assessed by body mass index (BMI) (reference group: BMI 18.5-24.9 kg/m^2):	Job strain	Cross-sectional associations adjusted for age, sex, and SES (13 studies):<br>Obese class I (30<=BMI<35 kg/m^2): 1.07 (1.02-1.12)<br>Obese classes II/III (BMI>=35 kg/m^2): 1.14 (1.01-1.28)<br>Prospective associations adjusted for age,

	Publication date	Ref number	Review / guidelines	Design(s) of primary studies	Other inclusion criteria	Number of primary studies	Outcome(s)	Exposure(s)	Main results: Pooled estimate(s) (95% CI)
									sex, and SES (4 studies):<br>Obesity (BMI>=30 kg/m^2):<br>0.99 (0.88-1.12)
Nyberg	2013	59	IPD-Work Consortium	Cross-sectional		8	Cardiovascular risk factors	Job strain	Adjusted for age, sex, and SES:<br>Hypertension<br>0.99 (0.93-1.04)<br>Diabetes<br>1.29 (1.11–1.51)<br>Smoking<br>1.14 (1.08–1.20)<br>High alcohol use<br>1.06 (0.99-1.14)<br>Physically inactive<br>1.34 (1.26–1.41)<br>Obesity<br>1.12 (1.04–1.20)<br>Framingham risk score >=20%<br>1.13 (1.03–1.25)
Palmer<br><br>Not in the extraction	2013	60	Literature review	All	1966- Dec. 2011<br>Abstract in English	86	Pregnancy outcomes	Long working hours ≥40 hours a week (reference group: <40 hours a week)	--- Preterm delivery (17 studies):<br>1.23 (1.13-1.34)<br>--- Small for gestational age (8 studies):<br>1.04 (0.94-1.16)
Rönnblad	2019	61	Literature review<br>PRISMA<br>MOOSE<br>MECIR	Longitudinal	Jan. 2000-Sept. 2017<br>Publications in a peer-reviewed journal<br>English, Danish, Norwegian, Swedish language<br>European Economic Area (European Union, Iceland, Lichtenstein and Norway), Switzerland, Australia, New Zealand, USA and Canada	10	Mental health outcomes	Job insecurity	Stratified or adjusted for gender, age and SES:<br>Depressive symptoms (5 studies):<br>1.61 (1.29-2.00), heterogeneity: I^2=44%<br>Anxiety (3 studies):<br>1.77 (1.18-2.65), heterogeneity: I^2=52%<br>Psychotropic drug use (2 studies):<br>1.30 (1.09-1.56), heterogeneity: I^2=0%

	Publication date	Ref number	Review / guidelines	Design(s) of primary studies	Other inclusion criteria	Number of primary studies	Outcome(s)	Exposure(s)	Main results: Pooled estimate(s) (95% CI)
Rudkjoebing	2020	62	Literature review PRISMA	Cohort, case-control, cross-sectional	Up to May 2018 Publications in a peer-reviewed journal English language	24	Depressive and anxiety disorder, depressive symptoms, anxiety symptoms, burnout, and disturbed sleep	Violence at the workplace defined as being exposed to direct aggressive physical assault or to threats of physical violence	Stratified or adjusted for gender, age and SES:<br>Depression (4 studies):<br>1.42 (1.31-1.54), heterogeneity: I^2=0%<br>Depressive symptoms (8 studies):<br>2.33 (1.71-3.17), heterogeneity: I^2=42%<br>Anxiety symptoms (3 studies):<br>2.40 (0.78-7.36), heterogeneity: I^2=90%<br>Burnout (6 studies):<br>1.60 (1.25-2.05), heterogeneity: I^2=57%<br>Sleep impairment (3 studies):<br>1.49 (1.14-1.96), heterogeneity: I^2=0%
Rugulies	2017	63	Literature review PRISMA	Prospective cohort	Up to Oct. 2016 Publications in a peer-reviewed journal English, German, Danish, Norwegian, Swedish, French, or Spanish language	8	Depressive disorders	Effort-reward imbalance (ERI)	Least adjusted:<br>1.68 (1.40–2.01), heterogeneity: I^2=60.2%
Stansfeld	2006	64	Literature review MOOSE	Longitudinal	1994–2005 English language Industrially established, market economy countries (Europe, North America, New Zealand, Australia, Japan, and Russia)	11	Common mental disorders	Low decision latitude (3 studies)<br><br>Job demands (5 studies)<br><br>Job strain (2 studies)<br><br>Low social support (5 studies)<br><br>Effort-reward imbalance (2 studies)<br>Job insecurity (2 studies)	Most highly adjusted:<br>1.226 (1.084-1.386), heterogeneity: I^2=44.2%<br>1.393 (1.152-1.685), heterogeneity: I^2=92.0%<br>1.815 (1.062-3.101), heterogeneity: I^2=92.0%<br>1.321 (1.213-1.438), heterogeneity: I^2=92.0%<br>1.844 (1.449-2.345), heterogeneity: I^2=92.0%<br>1.330 (1.062-1.666), heterogeneity: I^2=92.0%
Steptoe<br>Not in the extraction<br>Update of Kivimäki	2012	65	Literature review	Prospective	Up to Dec. 2011	17	Coronary heart disease	Job strain	Adjusted for age and sex:<br>1.4 (1.2-1.6), heterogeneity: I^2<8%

	Publication date	Ref number	Review / guidelines	Design(s) of primary studies	Other inclusion criteria	Number of primary studies	Outcome(s)	Exposure(s)	Main results:<br>Pooled estimate(s) (95% CI)
SJWEH 2006									
Sui	2016	66	Literature review<br>PRISMA<br>MOOSE	Prospective cohort	Before Sept. 2014<br>English language	7	Type 2 diabetes	Job demands (3 studies)<br><br>Low decision latitude (3 studies)<br><br>Job strain (6 studies)	Most fully adjusted:<br>0.94 (0.72–1.23), heterogeneity: I^2=52.0%<br>1.16 (0.85–1.58), heterogeneity: I^2=59.0%<br>1.12 (0.95–1.32), heterogeneity: I^2=61.0%
Szerencsi	2012	67	Literature review	Cohort, case-control, cross-sectional	Up to Feb. 2009<br>No restriction for language	71	Cardiovascular disease (CVD)	Job strain (46 studies)<br>High job demands (37 studies)<br>Low decision latitude (43 studies)	1.30 (1.14–1.46)<br>1.05 (0.97–1.14)<br>1.14 (1.05–1.23)
Taouk	2020	68	Literature review<br>PRISMA	Cohort	Up to the end of 2017	18	Coronary heart disease mortality	Low job control (5 studies)<br>High job demands (4 studies)<br><br>Job strain (7 studies)	Minimally-adjusted:<br>1.50 (1.42-1.58), heterogeneity: I^2=0.0%<br>0.89 (0.61-1.29), heterogeneity: I^2=69.4%<br>1.31 (0.91-1.88), heterogeneity: I^2=55.2%
Theorell<br><br>Not in the extraction	2015	69	Literature review	Prospective, case-control	1990-June 2013<br>Europe, North America, Australia and New Zealand<br>n>100<br>Results adjusted for age and adjustment or stratification for gender<br>English language	59	Depressive symptoms	Low decision latitude (13 studies)<br>Job strain (11 studies)<br>Bullying (3 studies)	Adjusted for age and gender - Least adjusted:<br>1.37 (1.30-1.47)<br>1.74 (1.53-1.96)<br>2.82 (2.21-3.59)
van der Molen<br><br>Update of Nieuwenhuijsen OM 2010	2020	70	Literature review<br>PRISMA	Prospective cohort	2008-Aug. 2019 + eligible studies before 2008 retrieved from the systematic review by Nieuwenhuijsen et al. 2010	17	(i) a stress-related disorder diagnosis following a clinical anamnesis, (ii) a high level of stress	Effort-reward imbalance (6 studies)<br>Low procedural justice (5 studies)<br>Low relational justice (5 studies)<br>Job demands (8 studies)<br>Low coworker support (4 studies)<br>Low supervisor support (5 studies)	1.91 (1.70-2.15), heterogeneity: I^2=48%<br><br>1.74 (1.62-1.86), heterogeneity: I^2=0%<br>1.55 (1.44-1.67), heterogeneity: I^2=0%<br>1.56 (1.41-1.72), heterogeneity: I^2=49%<br>1.29 (1.17-1.43), heterogeneity: I^2=0%<br>1.27 (1.16-1.38), heterogeneity: I^2=0%

	Publication date	Ref number	Review / guidelines	Design(s) of primary studies	Other inclusion criteria	Number of primary studies	Outcome(s)	Exposure(s)	Main results: Pooled estimate(s) (95% CI)
							complaints as assessed in a score above a cut-off point on a validated questionnaire for fatigue, stress or non-specific mental ill-health or (iii) absenteeism from work due to stress problems	Low support (2 studies)<br>Emotional demands (2 studies)<br>Low decision authority (2 studies)<br>Low decision latitude (2 studies)<br>Low skill discretion (2 studies)	1.41 (1.18-1.69), heterogeneity: I^2=10%<br>1.58 (1.35-1.84), heterogeneity: I^2=0%<br>1.34 (1.20-1.49), heterogeneity: I^2=0%<br>1.07 (0.92-1.25), heterogeneity: I^2=0%<br>1.11 (0.94-1.32), heterogeneity: I^2=0%
Van Melick	2014	71	Literature review PRISMA	Prospective or retrospective cohort, case-control, cross-sectional	1990-Nov.2013 English, German, French or Spanish language	13	Preterm birth	Long working hours >40 h/week (11 studies)	Most adjusted:<br>1.25 (1.01-1.54), heterogeneity: I^2=62%
Virtanen	2012	72	Literature review MOOSE	Prospective cohort, case-control, cross-sectional	Up to March 2011 Peer-reviewed studies English language	12	Coronary heart disease	Long working hours	Minimally adjusted and SES-adjusted (7 studies):<br>2.06 (1.55-2.74)
Virtanen	2013	73	Literature review MOOSE IPD-Work Consortium	Prospective cohort	Up to Oct. 2012 English language	17	Coronary heart disease	Job insecurity	Age-adjusted:<br>1.32 (1.09-1.59), heterogeneity: I^2=40.7%
Virtanen	2015	74	Literature review MOOSE IPD-Work Consortium	Cross-sectional, case-control, prospective	Up to April 2014 Peer-reviewed studies English language	81	Alcohol use	Long working hours	Cross-sectional association (61 studies):<br>--- alcohol use: 1.11 (1.05-1.18), heterogeneity: I^2=72%<br>--- risky alcohol use: 1.14 (0.89-1.47), heterogeneity: I^2=69%<br>Prospective association for new onset risky alcohol use (20 studies):<br>1.12 (1.04-1.20), heterogeneity: I^2=0%

	Publication date	Ref number	Review / guidelines	Design(s) of primary studies	Other inclusion criteria	Number of primary studies	Outcome(s)	Exposure(s)	Main results: Pooled estimate(s) (95% CI)
Virtanen<br><br>Update of Kivimäki L 2015	2018	75	Literature review<br>IPD-Work Consortium	Prospective cohort	Up to June 2018	23 (CHD)<br>15 (Stroke)	Coronary heart disease and stroke	Long working hours	Adjusted for age, sex, and SES:<br>--- coronary heart disease: 1.12 (1.03-1.21), heterogeneity: I^2=0%<br>--- stroke: 1.21 (1.01-1.45), heterogeneity: I^2=15.2%
Virtanen<br><br>Not in the extraction	2005	76	Literature review	Prospective, retrospective and cross-sectional	Up to Oct. 2003<br>Peer-reviewed studies<br>English, French, Spanish,or Italian language<br>Exclusion of studies that only focused on part-time, and those limited to job insecurity	27	Health outcomes including musculoskeletal disorders and psychological morbidity	Temporary employment	Musculoskeletal disorders (6 studies): 1.24 (0.69–2.22), heterogeneity: Q=484.19, P<0.001<br>Psychological morbidity (7 studies): 1.25 (1.14–1.38), heterogeneity: Q=32.91, P=0.012
Virtanen	2020	77	IPD-Work Consortium	Prospective cohort	Europe, North America, and Australia	19	Onset of overweight/ obesity (BMI $\geq$ 25 kg/m^2)	Long working hours (reference group: 35-40 h/week)	Adjusted for age, sex, and SES:<br>Working hours 41-48 h/week: 1.07 (1.02-1.12), heterogeneity: I^2=0.0%<br>Working hours 49-54 h/week: 1.09 (1.03-1.16), heterogeneity: I^2=0.0%<br>Working hours $\geq$ 55 h/week: 1.17 (1.08-1.27), heterogeneity: I^2=38.0%<br>Dose-response relation between longer working hours and the risk of increasing body weight (P for trend <0.0001 in meta-regression)
Virtanen	2018	78	Literature review<br>PRISMA<br>IPD-Work Consortium	Prospective cohort	Up to Jan. 2017<br>English language<br>Follow-up of 1-5 years	28	Depressive symptoms	Long working hours (defined as working ≥55 hours per week in most studies)	Adjustement for age, sex, SES, and marital status (unpublished studies) and closest to that set of adjustments (published studies):<br>1.14 (1.03-1.25), heterogeneity: I^2=45.1%
Watanabe	2016	79	Literature review<br>PRISMA	Prospective (cohort, nested case-control)	Up to July 2016	7	Depressive disorders clinically diagnosed or assessed by a structured interview	Overtime work (defined as at least >40 hours a week)	Adjusted for demographic covariates: 1.08 (0.83-1.39), heterogeneity: I^2=16.7%

	Publication date	Ref number	Review / guidelines	Design(s) of primary studies	Other inclusion criteria	Number of primary studies	Outcome(s)	Exposure(s)	Main results: Pooled estimate(s) (95% CI)
Watanabe	2018	80	Literature review PRISMA MOOSE	Longitudinal or prospective cohort	Up to 2016 English or Japanese language	8	Metabolic syndrome	Job strain (4 studies)	Adjusted by demographic variables (e.g. age, sex, education and marital status) and lifestyle variables (e.g. smoking, physical activity and sleep): 1.75 (1.09–2.79)
Wong	2019	81	Literature review	Cross-sectional, case-control, prospective cohort	1998-2018	46	Health outcomes	Long working hours	Cardiovascular heart diseases (11 studies): 1.539 (1.324-1.789)<br>Type 2 diabetes (2 studies): 0.855 (0.497-1.472)<br>Anxiety (2 studies): 1.308 (1.041-1.644)<br>Depressive symptoms (7 studies): 1.489 (1.220-1.817)<br>Heavy drinking (5 studies): 1.083 (0.943-1.244)<br>Physical inactivity (4 studies): 1.234 (1.002-1.520)<br>Smoking (4 studies): 1.055 (0.890-1.251)<br>Unhealthy food habits (3 studies): 0.990 (0.796-1.230)<br>Poor sleep quality (2 studies): 1.276 (1.128-1.444)<br>Short sleep duration (4 studies): 1.909 (1.502-2.427)<br>Sleep disturbance (3 studies): 1.395 (1.052-1.850)
Xu	2015	82	Literature review MOOSE	Prospective cohort	Up to Oct. 2014 No restriction on language	14	Coronary heart disease	High strain, passive and active job (reference group: low strain)<br>Low control, high demands	Multivariate-adjusted:<br>High-strain (11 studies): 1.26 (1.12–1.41), heterogeneity: I^2=23%<br>Passive job (9 studies): 1.14 (1.02–1.29), heterogeneity: I^2=0%<br>Active job (10 studies): 1.09 (0.97–1.22), heterogeneity: I^2=0%<br>Low control (11 studies): 1.06 (0.93–1.19), heterogeneity: I^2=54%<br>High demand (9 studies): 1.13 (0.97–1.32), heterogeneity: I^2=63%
Yang	2019	83	Literature review MOOSE	Case–control, cohort	Up to June 2018 No restriction on language	17	Cancer (any cancer, lung, colorectal,	Job strain	--- any cancer (13 studies): 1.06 (0.96–1.16), heterogeneity: I^2=1.9%<br>--- lung cancer (13 studies):

	Publication date	Ref number	Review / guidelines	Design(s) of primary studies	Other inclusion criteria	Number of primary studies	Outcome(s)	Exposure(s)	Main results: Pooled estimate(s) (95% CI)
							esophagus, prostate, breast)		1.16 (0.89–1.50), heterogeneity: I^2=0.0%<br>--- colorectal cancer (12 studies):<br>1.16 (0.90–1.48), heterogeneity: I^2=0.0%<br>--- prostate cancer (10 studies):<br>0.87 (0.69–1.10), heterogeneity: I^2=0.0%<br>--- breast cancer (12 studies):<br>1.06 (0.90–1.24), heterogeneity: I^2=12.8%
Yang	2018	84	Literature review PRISMA	Cross-sectional, prospective	1980-Jan. 2014<br>English or Japanese language<br>Europe and Asia	17	Insomnia symptoms	Effort-reward imbalance (5 studies)<br>High demand-low control (7 studies)<br>High demand (4 studies)<br>Low control (3 studies)<br>Low social support (3 studies)<br>Work-family conflict (2 studies)	2.63 (1.22–5.69), heterogeneity: I^2=95.1%<br>1.23 (1.14-1.34), heterogeneity: I^2=34.4%<br>1.35 (1.20–1.51), heterogeneity: I^2=29.0%<br>1.12 (0.99-1.26), heterogeneity: I^2=0.0%<br>1.67 (1.11–2.52), heterogeneity: I^2=61.3%<br>2.32 (1.53–3.51), heterogeneity: I^2=80.5%
Zhu	2020	85	Literature review MOOSE	Cross-sectional, case-control, cohort	Up to June 2019<br>English language	29	Weight-related outcomes (weight gain/BMI increase, BMI ≥ 25 kg/m^2, and BMI ≥ 30 kg/m^2)	Long working hours (defined as at least >40 hours a week)	All designs (29 studies):<br>1.13 (1.07-1.19), heterogeneity: I^2=75.7%

IPD-Work consortium studies with literature review were underlined in orange and those without literature review were underlined in yellow

Supplementary Table S1 (part 2). Description of the included reviews

First author	Publication date	Ref. number	Gender differences	Results from other subgroup/sensitivity analyses	Adjustment variables of main results	Statistical methods for pooled estimates	Publication bias assessment	Quality assessment of primary studies and/or evidence
Amiri	2020	14	Men (9 studies): 1.38 (1.09-1.75), heterogeneity: I^2=52.1%<br><br>Women (10 studies): 1.28 (1.01-1.63), heterogeneity: I^2=65.8%		None or any adjustment	Random effects model	Yes<br>No evidence of publication bias according to Begg's test and Egger's test	Yes<br>Effective Public Health Practice Project (EPHPPC) Quality Assessment Tool
Aronsson<br><br>Not in the extraction	2017	15			At least age and gender	Fixed effects model	No	Yes<br>GRADE
Babu	2014	16		Case-control (3 studies):<br>2.88 (1.63-5.09)<br>Cohort (6 studies):<br>1.24 (1.09-1.41)	Any adjustment	Random effects model	Yes<br>Minimal publication bias based on funnel plots	Yes
Bonde	2013	17	Not applicable	Studies of higher methodological quality (3 studies): 1.17 (0.80-1.71), heterogenity: Q=9.60, p=0.008	Any adjustment	Fixed effects model	Yes<br>Potential for overestimation of risks through publication bias based on funnel plots	Yes
Bonzini	2007	18	Not applicable	Studies of higher methodological quality (5 studies): 1.20 (0.98-1.47), heterogeneity: Q=1.32, p=0.86	Any adjustment	Random effects model	Yes<br>Funnel plots suggested a degree of publication bias (smaller studies increasingly more positive)	Yes
Cai	2019	19	Not applicable	Adjusted results:<br>--- Preterm delivery (6 studies):<br>1.25 (1.01-1.55), heterogeneity: I^2=0%<br>--- Miscarriage (5 studies):<br>1.73 (1.27-2.35), heterogeneity: I^2=0%<br>--- Preeclampsia (1 study):<br>1.04 (0.48-2.26), heterogeneity: not applicable	None or any adjustment	Random effects model	Yes<br>No evidence of publication bias based on funnel plots	Yes<br>Joanna Briggs Institute Critical Appraisal of Evidence Effectiveness tool, GRADE

First author	Publication date	Ref. number	Gender differences	Results from other subgroup/sensitivity analyses	Adjustment variables of main results	Statistical methods for pooled estimates	Publication bias assessment	Quality assessment of primary studies and/or evidence
				--- Gestational hypertension (1 study):<br>0.43 (0.20-0.91), heterogeneity: not applicable<br>--- Small for gestational age (5 studies):<br>1.76 (0.96-3.21), heterogeneity: I^2=76%<br>--- Low birth weight (3 studies):<br>1.37 (0.89-2.11), heterogeneity: I^2=0%<br><br>Unadjusted results:<br>--- Preterm delivery (19 studies):<br>1.21 (1.09-1.34), heterogeneity: I^2=40%<br>--- Miscarriage (5 studies):<br>1.22 (0.87-1.70), heterogeneity: I^2=86%<br>--- Preeclampsia (4 studies):<br>1.32 (0.70-2.49), heterogeneity: I^2=87%<br>--- Gestational hypertension (4 studies):<br>1.11 (0.86-1.43), heterogeneity: I^2=40%<br>--- Small for gestational age (7 studies):<br>1.06 (0.97-1.17), heterogeneity: I^2=0%<br>--- Low birth weight (3 studies):<br>1.42 (1.00-2.02), heterogeneity: I^2=18%<br><br>Test for differences between adjusted and unadjusted results:<br>--- Preterm delivery: p=0.77<br>--- Miscarriage: p=0.13<br>--- Preeclampsia: p=0.64<br>--- Gestational hypertension: p=0.02<br>--- Small for gestational age: p=0.11<br>--- Low birth weight: p=0.89				
Cosgrove	2012	20		Unadjusted or least adjusted versus most adjusted models	Most adjusted models	Random effects model	No	Yes
Descatha	2020	21	No gender difference	No difference according to WHO region, age, SES, and type of stroke	Adjusted for at least age, sex, and SES (except 2 cohort studies and 1 case-control study)	Random effects model	Yes<br>No serious concerns for publication bias based on funnel plots	Yes<br>Modified version of the Navigation Guide quality of evidence assessment approach based on GRADE
Dragano	2017	22	Men, adjusted for age and sex:<br>1.19 (1.02-1.40)<br>Women, adjusted for age and sex:	Adjusted for age and sex:<br>1.16 (1.01-1.34), heterogeneity: I^2=0%	Adjusted for age, sex, and SES	Random effects model	No	No

First author	Publication date	Ref. number	Gender differences	Results from other subgroup/sensitivity analyses	Adjustment variables of main results	Statistical methods for pooled estimates	Publication bias assessment	Quality assessment of primary studies and/or evidence
			1.08 (0.78-1.50)<br>No gender difference	Adjusted for age, sex, SES and lifestyle factors: 1.18 (1.02-1.37)<br><br>Adjusted for age, sex, SES, lifestyle factors, and job strain: 1.16 (1.00-1.35)<br><br>After exclusion of the first 3 years of follow-up, adjusted for age and sex: 1.21 (1.03-1.41), heterogeneity: I^2=0%<br><br>No difference according to age, SES, and ERI measure				
Duchaine	2020	23			Adjusted for age, sex, and SES or most of them	Random effects model	Yes<br>No publication bias was found in the funnel plots for job strain, psychological demands, or social support. Not enough studies to assess publication bias for the other psychosocial work stressors.	Yes<br>Risk of Bias in Non-Randomized Studies–Interventions (ROBINS-I) tool
Ferrie	2016	24	Adjustment for age, sex, SES, obesity, physical activity, alcohol use and smoking:<br>Men: 1.14 (0.99-1.31)<br>Women: 1.12 (0.96-1.32)<br>No gender difference	Adjusted for age and sex (19 studies): 1.19 (1.09–1.30), heterogeneity: I^2=24%<br><br>Adjusted for age, sex, SES and obesity, physical activity, alcohol use, and smoking:<br>--- all studies (15 studies): 1.12 (1.01–1.24)<br>--- high-quality studies: 1.19 (1.04–1.35)<br>--- poor-quality studies: 1.00 (0.84-1.20)<br><br>No difference according to study	Adjusted for age, sex, and SES	Fixed effects model because no significant heterogeneity between the study-specific estimates	No	Yes<br>Cochrane risk-of-bias tool for cohort studies

First author	Publication date	Ref. number	Gender differences	Results from other subgroup/sensitivity analyses	Adjustment variables of main results	Statistical methods for pooled estimates	Publication bias assessment	Quality assessment of primary studies and/or evidence
				quality, age, SES, method of diabetes diagnosis, and study location				
Fransson	2012	25	Cross-sectional associations stratified by gender and adjusted for sex, age, SES and smoking:<br>Men<br>--- high strain: 1.36 (1.28-1.44)<br>--- passive job: 1.27 (1.21-1.34)<br>--- active job: 1.05 (1.00-1.11)<br>Women<br>--- high strain: 1.28 (1.21-1.35)<br>--- passive job: 1.27 (1.21-1.34)<br>--- active job: 1.08 (1.02-1.14)<br>No gender difference	Cross-sectional associations adjusted for sex, age, SES, and smoking (10 studies):<br>--- high strain: 1.26 (1.15-1.38), heterogeneity: I^2=77.5%<br>--- passive job: 1.21 (1.11-1.31), heterogeneity: I^2=76.7%<br>--- active job: 1.06 (1.00-1.12), heterogeneity: I^2=55.6%<br><br>No differences according to age, SES, smoking status, or time of the study	Cross-sectional analyses: adjusted for sex and age<br><br>Prospective analyses: adjusted for sex, age, SES, and smoking	Cross-sectional data: 2-stage random effects model, subgroup analyses using 1-stage individual-level mixed-effects logistic regression model<br>Prospective data: 1-stage individual-level meta-analysis using mixed-effects logistic regression model	No	No
Fransson	2015	26		Age- and sex-adjusted results:<br>--- ischemic stroke: 1.24 (1.05-1.47), heterogeneity: I^2=2.4%<br>--- hemorrhagic stroke: 1.01 (0.75-1.36), heterogeneity: I^2=11.7%	Age- and sex-adjusted or age-, sex-, and SES adjusted	Random effects model	No	No
Hauke	2011	27			The most adjusted effect sizes available in each study	Random effects model	No	Yes
Heikkila<br><br>Not in the extraction	2016	28		Adjusted for age, sex, SES, shift work and night-time work:<br><br>Working hours 41-48 h/week:<br>--- any cancer (12 studies):	Adjusted for age and sex	Random effects model	No	No

First author	Publication date	Ref. number	Gender differences	Results from other subgroup/sensitivity analyses	Adjustment variables of main results	Statistical methods for pooled estimates	Publication bias assessment	Quality assessment of primary studies and/or evidence
				0.97 (0.87-1.07), heterogeneity: I^2=18.1%<br>--- colorectal cancer (10 studies):<br>1.03 (0.75-1.40), heterogeneity: I^2=0%<br>--- lung cancer (10 studies):<br>0.96 (0.64-1.44), heterogeneity: I^2=0%<br>--- breast cancer (10 studies):<br>0.91 (0.70-1.17), heterogeneity: I^2=8.2%<br>--- prostate cancer (9 studies):<br>0.88 (0.69-1.13), heterogeneity: I^2=4.9%<br><br>Working hours 49-54 h/week:<br>--- any cancer (11 studies):<br>1.07 (0.94-1.21), heterogeneity: I^2=0%<br>--- colorectal cancer (8 studies):<br>1.40 (0.93-2.11), heterogeneity: I^2=0%<br>--- lung cancer (5 studies):<br>0.82 (0.40-1.70), heterogeneity: I^2=0%<br>--- breast cancer (9 studies):<br>0.85 (0.55-1.31), heterogeneity: I^2=7.8%<br>--- prostate cancer (10 studies):<br>1.29 (0.97-1.71), heterogeneity: I^2=0%<br><br>Working hours ≥ 55 h/week:<br>--- any cancer (10 studies):<br>0.93 (0.81-1.06), heterogeneity: I^2=0%<br>--- colorectal cancer (7 studies):<br>1.03 (0.57-1.89), heterogeneity: I^2=25.9%<br>--- lung cancer (7 studies):<br>0.72 (0.37-1.40), heterogeneity: I^2=0%<br>--- breast cancer (6 studies):<br>1.49 (1.05-2.11), heterogeneity: I^2=0%<br>--- prostate cancer (9 studies):<br>1.07 (0.65-1.77), heterogeneity: I^2=33.0%				
Heikkila	2013	29		Adjusted for age, sex, SES, BMI, smoking, and alcohol intake:<br>--- any cancer (12 studies):<br>0.97 (0.90-1.04), heterogeneity: I^2=0%<br>--- colorectal cancer (12 studies):	Adjusted for age and sex	Random effects model	No	No

First author	Publication date	Ref. number	Gender differences	Results from other subgroup/sensitivity analyses	Adjustment variables of main results	Statistical methods for pooled estimates	Publication bias assessment	Quality assessment of primary studies and/or evidence
				1.16 (0.90-1.48), heterogeneity: I^2=0%<br>--- lung cancer (12 studies):<br>1.17 (0.88-1.54), heterogeneity: I^2=0%<br>--- breast cancer (11 studies):<br>0.97 (0.82-1.14), heterogeneity: I^2=0%<br>--- prostate cancer (10 studies):<br>0.86 (0.68-1.09), heterogeneity: I^2=0%<br><br>No evidence of associations between high strain, active job, passive job, versus low strain, and risk of overall cancer and of each cancer type				
Heikkila	2020	30	Men: 1.59 (1.12-2.28), heterogeneity: I^2=44.8%<br>Women: 1.15 (0.80-1.66), heterogeneity: I^2=0.0%<br>No gender difference	Unadjusted: 1.25 (1.04-1.50), heterogeneity: I^2=0.0%<br><br>Adjustment for age, sex, and lifestyle-related covariates: 1.41 (1.11-1.80), heterogeneity: I^2=26.9%<br><br>No difference according to baseline smoking status<br><br>Significant difference according to SES (p=0.0.46):<br>--- low SES: 1.30 (0.97-1.76), heterogeneity: I^2=5.3%<br>--- intermediate SES: 1.08 (0.77-1.50), heterogeneity: I^2=0.0%<br>--- high SES: 2.77 (1.35-5.71), heterogeneity: I^2=10.5%	Adjustment for age and sex	Random effects model	No	No
Heikkila	2012	31	Cross-sectional associations based on the pooled individual-level data, stratified by gender and adjusted for age and SES (10 studies), with job strain as outcome:<br>--- men: smokers: 1.13 (1.07-1.20)<br>--- women: smokers: 1.06 (1.00-1.11)<br>No gender difference	Cross-sectional associations in subgroups (10 studies, pooled individual-level data): significant small differences in the associations according to age and SES groups	Adjusted for age, sex and SES	Random effects model	No	No
Heikkila	2012	32	Cross-sectional associations based on the pooled individual-level data, stratified by gender and adjusted for age and SES (8 studies), with job	Cross-sectional associations in subgroups (8 studies, pooled individual-level data): lower odds of job strain for those aged 50 or older, no clear pattern	Adjusted for age, sex and SES	Random effects model	No	No

First author	Publication date	Ref. number	Gender differences	Results from other subgroup/sensitivity analyses	Adjustment variables of main results	Statistical methods for pooled estimates	Publication bias assessment	Quality assessment of primary studies and/or evidence
			strain as outcome:<br>--- men: intermediate drinkers: 0.96 (0.87-1.07)<br>--- women: intermediate drinkers: 0.91 (0.81-1.03)<br>No gender difference<br>--- men: heavy drinkers: 1.17 (1.08-1.26)<br>--- women: heavy drinkers: 1.02 (0.93-1.13)<br>No gender difference	of the odds of job strain according to socioeconomic position				
Heikkila	2014	33		Adjusted for age, sex, SES, smoking, and BMI:<br>--- Crohn's disease: 0.83 (0.48-1.43), heterogeneity: I^2=0.0%<br>--- Ulcerative colitis: 1.06 (0.76-1.48), heterogeneity: I^2=19.7%	Age and sex-adjusted	Random effects model	No	No
Huang	2015	34	Men (3 studies): 1.26 (0.69–2.27)<br>Women (5 studies): 1.33 (1.04–1.69)<br>No gender difference	Significant association with high strain after exclusion of the only study with unadjusted results: 1.24 (1.01-1.53) for all stroke	None and any adjustment	Fixed effects model when no significant heterogeneity between studies, otherwise random-effects model	Yes<br>No evidence of publication bias according to funnel plots and Egger tests	Yes<br>Newcastle-Ottawa Quality Assessment Scale
Kang	2012	35	Men (6 studies): 1.37 (0.96-1.96)<br>Women (2 studies): not provided	Case-control (6 studies): 1.43 (1.00-2.03)<br>Cohort (5 studies): 1.41 (1.12-1.77)<br><br>Long working hours defined as up to 55 hours a week (9 studies): 1.29 (0.99-1.66)	Any adjustment	Random effects model when p<0.10 for heterogeneity between studies, otherwise fixed-effects model	Yes<br>No evidence of publication bias according to Egger's test	No
Kim	2016	36		Unadjusted model (1 study): 1.56 (1.40–1.73)<br><br>Adjusted model (5 studies):	None or any adjustment	Random effects model when expected significant	Yes<br>No evidence of publication bias according to	No

First author	Publication date	Ref. number	Gender differences	Results from other subgroup/sensitivity analyses	Adjustment variables of main results	Statistical methods for pooled estimates	Publication bias assessment	Quality assessment of primary studies and/or evidence
				1.23 (1.01–1.50), heterogeneity: I^2=83%<br><br>Higher ORs in Europe, younger participants, with an exposure-outcome time lag between 3 and 4 years		heterogeneity between studies, otherwise fixed-effects model	Begg'stest and Egger's test	
Kivimaki	2013	37		Adjusted for age, sex, cohort, and lifestyle factors: 1.21 (1.03–1.43)	Adjusted for age, sex, and cohort	Cox regression model using pooled individual-level data	No	No
Kivimaki<br><br>Update of Kivimaki E 2014	2015	38			-		No	No
Kivimaki<br><br>Idem Steptoe ARPH 2013	2014	39					No	No
Kivimaki<br><br>Not in the extraction	2018	40		Adjusted for age, sex, cohort, SES, smoking, high alcohol intake, body mass index, and leisure-time physical inactivity:<br>--- Venous thromboembolism: 1.5 (1.1-2.2)<br>--- Deep-vein thrombosis: 1.7 (1.1-2.6)<br>--- Pulmonary embolism: 1.4 (0.8-2.4)	Adjusted for age, sex, cohort, and SES	Prospective multi-cohort study from individual-level data	No	No
Kivimaki	2017	41	No gender difference	Adjusted for age, sex, SES, and lifestyle-related factors (7 studies): 1.41 (1.10-1.80), heterogeneity: I^2=0%<br><br>Adjusted for age, sex, SES, and pre-existing coronary heart disease at the time of atrial fibrillation diagnosis: 1.41 (1.12-1.78)<br><br>Adjusted for age, sex, and SES results stratified by method of outcome	Adjusted for age, sex and SES	Random effects model	No	No

First author	Publication date	Ref. number	Gender differences	Results from other subgroup/sensitivity analyses	Adjustment variables of main results	Statistical methods for pooled estimates	Publication bias assessment	Quality assessment of primary studies and/or evidence
				assessment:<br>--- Electronic health records (hospitalisation, death) (5 studies): 1.65 (1.03-2.66), heterogeneity: I^2=0%<br>--- Electronic health records (hospitalisation, death, drug reimbursement register) (3 studies): 1.32 (0.93-1.88), heterogeneity: I^2=0%<br>--- Electrocardiogram (1 study): 1.41 (0.93-2.14)<br><br>No difference according to age and SES groups				
Kivimaki	2015	42		Weight gain (4 studies), fixed-effect meta-analysis: 1.04 (0.99-1.09)<br><br>Obesity (4 studies), fixed-effect meta-analysis: 1.00 (0.89-1.13)	Adjusted for age, sex and SES	Fixed and random effects models	No	No
Kivimaki	2012	43	Men, adjusted for age and sex: 1.29 (1.13-1.48)<br>Women, adjusted for age and sex: 1.46 (1.07-1.99)<br>No gender difference	Adjusted for age and sex: 1.23 (1.10-1.37), heterogeneity: I^2<0.1%<br><br>Adjusted for age, sex, SES, and lifestyle factors (7 studies): 1.21 (1.03-1.44)<br><br>Adjusted for age, sex, SES, and Framingham score (4 studies): 1.42 (1.16-1.74)<br><br>Slightly stronger association after exclusion of CHD cases at the first 3 years and 5 years of follow-up in order to minimise reverse causality<br><br>Few differences in the association between studies from Nordic countries, continental Europe, and the UK<br><br>Significant association in all levels of SES<br><br>Comparison of age- and sex-adjusted	Adjusted for sex, age and SES	Random effects model	No	No

First author	Publication date	Ref. number	Gender differences	Results from other subgroup/sensitivity analyses	Adjustment variables of main results	Statistical methods for pooled estimates	Publication bias assessment	Quality assessment of primary studies and/or evidence
				results according to publication status<br>--- published: (3 studies) :<br>1.43 (1.15-1.77)<br>--- unpublished: (10 studies):<br>1.16 (1.02-1.32)<br><br>Age- and sex-adjusted results for job strain categories:<br>Passive: 1.12 (0.99-1.27)<br>Active: 1.06 (0.94-1.19)<br>High strain: 1.28 (1.11-1.48)				
Kivimaki	2015	44	Adjusted for age and SES:<br><br>--- Coronary heart disease<br>Men: 1.09 (0.94-1.26)<br>Women: 1.20 (0.89-1.63)<br>No gender difference<br><br>--- Stroke<br>Men: 1.29 (1.04-1.60)<br>Women: 1.63 (1.10-2.43)<br>No gender difference	High quality studies, adjusted for age, sex and SES:<br>--- coronary heart disease:<br>1.17 (0.99–1.39)<br>--- stroke: 1.32 (0.99–1.76), heterogeneity: I^2=0%<br><br>No significant difference according to age, SES, geographical region, method of outcome ascertainment, and publication status	Adjusted for age, sex, and SES	Random effects model	Yes<br>A funnel plot of published studies on long working hours and incident coronary heart disease did not provide evidence of publication bias	Yes<br>Cochrane risk-of-bias tool for cohort studies
Kivimaki	2015	45	Men: 0.98 (0.83-1.16)<br>Women: 1.16 (0.92-1.47)<br>No gender difference	No difference according to publication status:<br>--- published (4 studies):<br>1.18 (0.60–2.32), heterogeneity: I^2=76%<br>--- unpublished (19 studies):<br>1.05 (0.87–1.25), heterogeneity: I^2=47%<br><br>No significant difference according to method of diabetes ascertainment, length of follow-up, study location, age group, or obesity status<br><br>Significant differences according to SES group (adjusted for age and sex):<br>--- low: 1.29 (1.06-1.57), heterogeneity: I^2=0%<br>--- intermediate: 1.13 (0.88-1.44), heterogeneity: I^2=0%<br>--- high: 1.00 (0.80-1.25), heterogeneity: I^2=15%	Adjusted for age and sex	Random effects model	Yes<br>No difference according to publication status (published versus unpublished)	No

First author	Publication date	Ref. number	Gender differences	Results from other subgroup/sensitivity analyses	Adjustment variables of main results	Statistical methods for pooled estimates	Publication bias assessment	Quality assessment of primary studies and/or evidence
Kivimaki	2006	46		Multiple adjusted:<br>--- job strain (10 studies):<br>1.16 (0.94–1.43), heterogeneity: Q=15.4, p=0.12<br>--- effort-reward imbalance (4 studies):<br>2.05 (0.97–4.32), heterogeneity: Q=6.4, p=0.04<br>--- organizational injustice (2 studies):<br>1.47 (1.12–1.95), heterogeneity: not tested	Age- and gender-adjusted	Random effects model	Yes<br>Some evidence for publication bias as shown by funnel plot asymmetry	No
Kraatz	2013	47			Adjusted for at least one physical factor	Random effects model	No	Yes
Lang	2012	48				Random effects model	Yes<br>The statistical test for publication bias was significant for the lagged effect of low social support on lower extremity problems. Statistical Egger's tests provided no evidence for publication bias, for all other relationships	Yes
Li	2020	49	No gender difference<br><br>Results from cohort studies for working hours ≥ 55 h/week:<br><br>--- incidence (p=0.99)<br>Men: 1.20 (0.80-1.82)<br>Women: 1.21 (0.77-1.91)<br><br>--- mortality (p=0.99)	Working hours 41-48 h/week:<br>--- incidence (2 case-control studies):<br>0.26 (0.13-0.49), heterogeneity: I^2=0%<br><br>Working hours 49-54 h/week:<br>--- incidence (2 case-control studies):<br>0.23 (0.09-0.59), heterogeneity: I^2=0%<br><br>Working hours ≥ 55 h/week:	Adjusted for at least age, sex, and SES	Random effects model	Yes<br>No serious concerns for publication bias according to funnel plots	Yes<br>Navigation Guide, GRADE

First author	Publication date	Ref. number	Gender differences	Results from other subgroup/sensitivity analyses	Adjustment variables of main results	Statistical methods for pooled estimates	Publication bias assessment	Quality assessment of primary studies and/or evidence
			Men: 1.21 (0.97-1.52)<br>Women: 1.21 (0.77-1.91)	--- incidence (2 case-control studies): 0.74 (0.41-1.34), heterogeneity: I^2=0%<br><br>Stronger associations in lower SES subgroup (incidence p=0.05, mortality p=0.05)<br><br>No difference according to WHO region, outcome measurement, and risk of bias status				
Linton<br><br>Not in the extraction	2015	50			Least adjusted	Random effects model	No	Yes<br>GRADE
Madsen	2017	51	Sex-stratified results from unpublished studies:<br>Men (11 studies): 1.21 (0.92-1.60)<br>Women (11 studies): 1.30 (1.04-1.63)<br>No gender difference	High-quality published studies, adjusted for sex, age, marital status, and education: 1.78 (1.46–2.17)<br><br>Unpublished studies:<br>--- similar estimates across age and SES subgroups<br>--- after additional adjustment for baseline depressive symptoms (as a continuous score), the association disappeared: 1.03 (0.81–1.32)<br>--- using the four quadrants of job strain, the risk of clinical depression was increased in job strain and passive work groups compared with low strain	Any adjustment (published studies)<br>Adjusted for age, sex, cohabitation, and SES (unpublished studies)	Random effects model	No	Yes<br>Newcastle-Ottawa Quality Assessment Scale
Magnavita	2019	52			Any adjustment	Random effects model	Yes<br>Asymmetric funnel plot indicated the presence of publication bias	Yes<br>Newcastle-Ottawa scales (NOS, NOS-A)
Milner	2018	53	Some evidence of gender differences<br><br>Associations with suicide death in men only:<br>Low support: 1.09 (0.89-1.33), heterogeneity: I^2=98.6%<br>Low control: 1.29 (1.19-1.40),		Unadjusted	Random effects model	Yes<br>Potential publication bias	No

First author	Publication date	Ref. number	Gender differences	Results from other subgroup/sensitivity analyses	Adjustment variables of main results	Statistical methods for pooled estimates	Publication bias assessment	Quality assessment of primary studies and/or evidence
			heterogeneity: I^2=100%<br>Job demands: 1.16 (0.57-2.37), heterogeneity: I^2=88.5%					
Milner	2019	54	Gender did not influence the results	Study design, choice of outcome, and country of study did not influence the results<br><br>High-quality studies (quality score >=5): results in the same direction but reduced in significance	Any adjustment	Random effects model	Yes	Yes<br>Newcastle-Ottawa Quality Assessment Scale
Nielsen	2020	55	Cross-sectional studies:<br>--- Men: 3.04 (1.79-5.17), heterogeneity: I^2=81.0%<br>--- Women: 2.55 (1.50-4.33), heterogeneity: I^2=91.5%<br>No gender difference				Yes<br>No publication bias according to four indicators (funnel plot, Orwin's Fail-Safe N, Duval and Tweedie's trim and fill procedure, and Egger's test)	Yes<br>GRADE
Nieuwenhuijsen	2010	56	Low job control:<br>--- Men: 1.24 (1.09-1.41)<br>--- Women: 1.18 (0.97-1.44)<br><br>Low co-worker support:<br>--- Men: 1.27 (1.13-1.43)<br>--- Women: 1.18 (0.99-1.41)<br><br>Low supervisor support:<br>--- Men: 1.25 (1.05-1.49)<br>--- Women: 1.11 (0.96-1.30)		Any adjustment	Fixed effects models	No	Yes<br>All included studies were of high quality
Nyberg	2014	57	No gender difference<br>Men: 1.19 (1.06-1.34)<br>Women: 1.13 (1.00-1.28)	Adjusted for age, sex, SES, and lifestyle variables (BMI, physical activity, smoking status, and alcohol consumption): 1.11 (1.00-1.23)<br><br>After exclusion of the first 3 years of follow-up, adjusted for age, sex and SES: 1.15 (1.06-1.25)	Adjusted for age, sex, and SES	Fixed effects model	No	No

First author	Publication date	Ref. number	Gender differences	Results from other subgroup/sensitivity analyses	Adjustment variables of main results	Statistical methods for pooled estimates	Publication bias assessment	Quality assessment of primary studies and/or evidence
				No difference according to age and method of diabetes ascertainment				
Nyberg	2012	58	No gender difference in cross-sectional associations	Cross-sectional associations adjusted for age and sex (13 studies):<br>--- Obese class I (30<=BMI<35 kg/m²): 1.19 (1.13-25)<br>--- Obese classes II/III (BMI>=35 kg/m²): 1.30 (1.16-1.46)<br>--- Obesity (BMI>=30 kg/m²): 1.22 (1.16-1.28)<br><br>Cross-sectional associations:<br>- Further adjustment for smoking had no effect on the estimates<br>- No difference in cross-sectional associations in participants younger and older than 50 years of age<br>- Unchanged results when the analyses were performed separately for clinically measured versus self-reported BMI<br><br>Prospective associations:<br>Adjustment for the length of follow-up had essentially no effect on the longitudinal association estimates.	Adjusted for age, sex, and SES	Cross-sectional data: 2-stage random effects meta-analysis Subgroup differences and prospective associations: 1-stage meta-analysis pooling all available individual-level data into one data set	No	No
Nyberg	2013	59	No significant interaction between sex and job strain for diabetes		Adjusted for age, sex, and SES	Cross-sectional study individual-level data from the studies were pooled into one dataset to be analyzed using mixed effects linear and logistic regression models with	No	No

First author	Publication date	Ref. number	Gender differences	Results from other subgroup/sensitivity analyses	Adjustment variables of main results	Statistical methods for pooled estimates	Publication bias assessment	Quality assessment of primary studies and/or evidence
						the study as the random effect		
Palmer<br><br>Not in the extraction	2013	60	Not applicable	High-quality studies (excluding studies with a higher potential for bias or confounding, or which reported incompletely):<br>--- Preterm delivery (11 studies): 1.18 (1.05-1.33)<br>--- Small for gestational age (6 studies): 0.99 (0.88-1.11)		Fixed effects model when no significant heterogeneity between studies ($p>=0.10$), otherwise random-effects model	Yes<br>Risk estimates were lower in the largest and better studies, with outliers confined to small studies, suggesting publication bias	Yes
Rönnblad	2019	61		Dose-response associations for depressive symptoms and anxiety<br>Meta-analyses were reperformed using the most adjusted models provided in each study showed only marginal decreases in effect sizes and did not alter any conclusions	Stratified or adjusted for gender, age and SES	Random effects model	Yes<br>Funnel plots did not show significant skewedness	Yes<br>GRADE
Rudkjoebing	2020	62			Stratified or adjusted for gender, age and SES	Random effects model	Yes<br>Publication bias was consided as unlikely according to funnel plot	Yes
Rugulies	2017	63		All studies, based on the most adjusted risk estimates of each study:<br>1.49 (1.23–1.80), heterogeneity: $I^2=59.4\%$<br><br>The pooled estimates were similar in all stratified analyses according to study quality, ERI ascertainment, and depressive disorders ascertainment	Least adjusted	Random effects model	Yes<br>Funnel plot skewed to the right, which could be an indication of publication bias, Egger's test did not indicate any publication bias	Yes<br>Newcastle-Ottawa Quality Assessment Scale
Stansfeld	2006	64	Sex-stratified results for job demands:<br>--- Men: 1.55 (1.29-1.85), heterogeneity: $I^2=70.7\%$		Most highly adjusted	Random effects model	No	No

First author	Publication date	Ref. number	Gender differences	Results from other subgroup/sensitivity analyses	Adjustment variables of main results	Statistical methods for pooled estimates	Publication bias assessment	Quality assessment of primary studies and/or evidence
			--- Women: 1.34 (1.16-1.53), heterogeneity: I^2=0%<br><br>Sex-stratified results for low social support:<br>--- Men: 1.38 (1.20-1.60), heterogeneity: I^2=71.8%<br>--- Women: 1.20 (1.07-1.35), heterogeneity: I^2=0%					
Steptoe<br>Not in the extraction<br>Update of Kivimäki SJWEH 2006	2012	65			Adjusted for age and sex	Random effects model	No	No
Sui	2016	66	Sex-stratified results:<br>Job demands:<br>--- Men (3 studies): 0.82 (0.52-1.28), heterogeneity: I^2=72%<br>--- Women (3 studies):<br>1.12 (0.83-1.51), heterogeneity: I^2=0%<br><br>Low social support:<br>--- Men (3 studies): 0.88 (0.71-1.09), heterogeneity: I^2=0%<br>--- Women (3 studies):<br>1.64 (0.99-2.72), heterogeneity: I^2=0%<br><br>Job strain:<br>--- Men (4 studies): 0.88 (0.59-1.33), heterogeneity: I^2=78%<br>--- Women (4 studies):<br>1.22 (1.01-1.46), heterogeneity: I^2=28%<br><br>No gender difference for demands, support, and job strain in association with diabetes		Most fully adjusted	Random effects model	Yes<br>No publication bias was detected using a funnel plot	Yes<br>Newcastle-Ottawa Quality Assessment Scale
Szerencsi	2012	67		Differences according to study characteristics, including country of study, assessment methods for exposure		Random effects model	Yes<br>Visual inspection of the funnel plot	Yes

First author	Publication date	Ref. number	Gender differences	Results from other subgroup/sensitivity analyses	Adjustment variables of main results	Statistical methods for pooled estimates	Publication bias assessment	Quality assessment of primary studies and/or evidence
				and outcome, and some adjustment factors			indicates the presence of bias since smaller studies (less precision) showing no effects are missing. The Egger test confirms the finding of bias (P=0.004)	
Taouk	2020	68	Sex-stratified results:<br><br>Low job control<br>--- minimally adjusted analysis:<br>Men (3 studies): 1.36 (1.16-1.58), heterogeneity: I^2=0.0%<br>Women (1 study): 1.21 (0.83-1.76), heterogeneity: not applicable<br>--- multivariable-adjusted analysis<br>Men (2 studies): 1.16 (0.97-1.39), heterogeneity: I^2=0.0%<br>Women (1 study): no study<br><br>High job demands<br>--- minimally adjusted analysis:<br>Men (3 studies): 0.97 (0.55-1.72), heterogeneity: I^2=72.3%<br>Women (1 study): 0.56 (0.39-0.86), heterogeneity: not applicable<br><br>Job strain<br>--- minimally adjusted analysis:<br>Men (3 studies): 1.37 (0.75-2.50), heterogeneity: I^2=73.0%<br>Women (3 studies): 0.85 (0.53-1.37), heterogeneity: I^2=0.0%<br>--- multivariable-adjusted analysis<br>Men (1 study): 1.03 (0.75-1.41), heterogeneity: not applicable	Low job control, multivariable-adjusted analysis (6 studies): 1.23 (1.17-1.30), heterogeneity: I^2=0.0%<br><br>Job strain, multivariable-adjusted analysis: 1.26 (0.82-1.94), heterogeneity: I^2=35.0%<br><br>Excluding studies with low quality assessment resulted in attenuated pooled estimates and wider confidence intervals. However, the association between low job control and CHD mortality remained the same:<br>--- minimally adjusted analysis:<br>1.36 (1.19-1.56)<br>--- multivariable-adjusted analysis:<br>1.19 (1.01-1.40)	Minimally-adjusted	Random effects model	Yes<br>Funnel plots suggested some degree of asymmetry (publication bias), however the Egger's tests did not detect strong evidence of small study effects and risk of publication bias	Yes<br>Scottish Intercollegiate Guidelines Network (SIGN)

First author	Publication date	Ref. number	Gender differences	Results from other subgroup/sensitivity analyses	Adjustment variables of main results	Statistical methods for pooled estimates	Publication bias assessment	Quality assessment of primary studies and/or evidence
			Women (1 study): 0.84 (0.35-2.04), heterogeneity: not applicable					
Theorell<br><br>Not in the extraction	2015	69			Adjusted for age and gender<br>Least adjusted	Fixed effects models	Yes<br>No evidence of publication bias based on funnel plots	Yes<br>GRADE
van der Molen<br><br>Update of Nieuwenhuijsen OM 2010	2020	70	No gender difference in associations for:<br>- Effort-reward imbalance<br>- Low procedural justice<br>- Low relational justice<br>- High emotional demands<br>- Low decision authority		Any adjustment	Random effects model	Yes<br>Visual checking of the funnel plots suggested no serious publication bias for effort-reward imbalance, low procedural justice, low relational justice and low support (coworker, supervisor, or combined).<br>Publication bias could not be assessed for the other psychosocial work factors	Yes<br>Quality of included studies<br>Quality of evidence using the GRADE framework
Van Melick	2014	71	Not applicable	Case-control (3 studies):<br>1.08 (0.92-1.28), heterogeneity: I^2=0%<br>Cohort (8 studies): 1.35 (0.98-1.87), heterogeneity: I^2=69%<br>No difference between study design	Most adjusted	Random effects model	No	Yes<br>Scottish Intercollegiate Guidelines Network (SIGN)
Virtanen	2012	72	Only men (7 studies): 2.07 (1.51-2.85)<br><br>Women with or without men (5 studies): 1.43 (1.05-1.93)	Minimally adjusted (at least controlled for age and sex): 1.80 (1.42-2.29), heterogeneity: I^2=61.9%<br><br>Maximally adjusted (5 studies):<br>1.59 (1.23-2.07)	Minimally adjusted and SES	Random effects model	Yes<br>The funnel plot for minimally adjusted study results appeared	No

First author	Publication date	Ref. number	Gender differences	Results from other subgroup/sensitivity analyses	Adjustment variables of main results	Statistical methods for pooled estimates	Publication bias assessment	Quality assessment of primary studies and/or evidence
				Case-control (7 studies):<br>2.43 (1.81-3.26)<br><br>Prospective ohort (4 studies):<br>1.39 (1.12-1.72)<br><br>>50 hours/week or >10 hours/day (5 studies): 2.37 (1.56-3.59)<br><=50 hours/week or <=10 hours/day (5 studies): 1.41 (1.14-1.74)<br><br>No clear differences in estimates when the studies were stratified by geographic region			symmetric and the Egger's test was not significant. No evidence of publication bias	
Virtanen	2013	73	Sex-stratified results adjusted for age:<br>--- Men (15 studies): 1.24 (0.98-1.57), heterogeneity: I^2=43.7%<br>--- Women (13 studies):<br>1.47 (1.07-2.02), heterogeneity: I^2=37.3%<br>No gender difference in multivariable-adjusted analysis	Multivariable adjusted (additionally adjusted SES, smoking, alcohol use, physical activity, body mass index, hypertension, hypercholesterolaemia, and diabetes) (17 studies):<br>1.19 (1.00-1.42), heterogeneity: I^2=24.6%<br><br>No difference according to age, national unemployment rate, welfare regime, and job insecurity measure	Minimally adjusted: age	Random effects model	No	No
Virtanen	2015	74	No gender difference<br><br>Prospective association:<br>--- Men: 1.11 (0.98-1.15)<br>--- Women: 1.36 (1.10-1.68)	Cross-sectional association (alcohol use):<br>--- unpublished studies:<br>1.10 (1.04-1.18), heterogeneity: I^2=18%<br>--- published studies: 1.12 (1.02-1.22), heterogeneity: I^2=81%<br><br>Prospective association:<br>--- unpublished studies:<br>1.12 (1.01-1.25), heterogeneity: I^2=0%<br>--- published studies: 1.10 (0.66-1.84), heterogeneity: I^2=56%<br><br>No difference according to age, SES, geographical regions, sample type (population based *vs* occupational	Adjusted for sex, age, SES, and ethnicity (unpublished studies)<br>None or any adjustment (published studies)	Random effects model	Yes<br>The funnel plot for cross-sectional published studies appears symmetrical and Egger's test was not significant	No

First author	Publication date	Ref. number	Gender differences	Results from other subgroup/sensitivity analyses	Adjustment variables of main results	Statistical methods for pooled estimates	Publication bias assessment	Quality assessment of primary studies and/or evidence
				cohort), levels of risky alcohol use, or sample attrition rate				
Virtanen<br><br>Update of Kivimäki L 2015	2018	75			Adjusted for age, sex, and SES	Random effects model	No	No
Virtanen<br><br>Not in the extraction	2005	76			None or any adjustment	Random effects model	No	No
Virtanen	2020	77	Sex-stratified results:<br>--- Men (18 studies): 1.24 (1.09-1.41), heterogeneity: I^2=56.4%<br>--- Women (17 studies):<br>1.08 (0.97-1.20), heterogeneity: I^2=0.0%	Multivariable adjustment for chronic somatic disease, mental health and lifestyle (16 studies) for working hours ≥ 55 h/week: 1.12 (1.01-1.25), heterogeneity: I^2=45.4%<br><br>Sensitivity analyses in which cigarette smokers, those with chronic somatic diseases, and those with underweight were excluded, had little impact on the associations.<br><br>Similar results according to age, SES, or duration of follow-up	Adjusted for age, sex, and SES	Random effects model	No	No
Virtanen	2018	78	Sex-stratified results:<br>--- Men: 1.08 (0.97-1.20)<br>--- Women: 1.14 (1.00-1.29)<br>No gender difference	A significant difference was found for Asia versus other geographic regions (p=0.034): 1.50 (1.13–2.01) for Asia, 1.11 (1.00–1.22) for Europe, 0.97 (0.70–1.34) for North America, and 0.95 (0.70–1.29) for Australia.<br><br>There were two other characteristics with subgroup differences:<br>- published *vs* unpublished (p-values 0.075 and 0.018 using heterogeneity test and meta-regression respectively): 1.35 (1.07-1.71) *vs* 1.08 (1.00-1.16),<br>- population-based *vs* occupational cohort (P-values 0.048 and 0.092 respectively):<br>1.07 (0.96-1.20) *vs* 1.34 (1.10-1.62)	Adjustement for age, sex, SES, and marital status (unpublished studies) and closest to that set of adjustments (published studies)	Random effects model	Yes<br>The Egger's test did not show evidence for publication bias	Yes<br>Cochrane risk-of-bias tool for cohort studies

First author	Publication date	Ref. number	Gender differences	Results from other subgroup/sensitivity analyses	Adjustment variables of main results	Statistical methods for pooled estimates	Publication bias assessment	Quality assessment of primary studies and/or evidence
				No difference according to age, SES, publication status, study baseline year, study quality, follow-up, response rate at baseline, loss to follow-up, outcome type, population prevalence of symptoms at baseline, and population onset of symptoms at follow-up.				
Watanabe	2016	79	Sex-stratified results:<br>--- Men (3 studies): 0.79 (0.49-1.26), heterogeneity: I^2=0.0%<br>--- Women (1 study): 2.20 (1.10-4.40), heterogeneity: not applicable	Meta-analyses stratified by geographical areas indicated that the pooled RRs for Asian countries (Japan only) and for the Western countries were similar: 1.07 (0.73-1.56) and 1.05 (0.68-1.61) respectively.<br><br>In the sensitivity analysis using the overtime criteria of working 50 hours or more per week, the pooled RR was 1.24 (0.88-1.75).<br><br>Follow-up length and publication year were not significantly correlated with pooled estimates.	Adjusted for demographic covariates	Random effects model	Yes<br>A funnel plot indicated a relatively smaller number of studies with greater SE (ie, with a smaller sample size) reporting greater RRs. The Egger's test was not significant.	No
Watanabe	2018	80			Adjusted by demographic variables (e.g. age, sex, education and marital status) and lifestyle variables (e.g. smoking, physical activity and sleep)	Random effects model	No	Yes<br>Newcastle-Ottawa Quality Assessment Scale
Wong	2019	81	No gender differences	Study of differences according to diagnosis method, study design, cut-off point for long working hours, working class, country of origin, and health measure	None or any adjustment	Random effects model	No	No
Xu	2015	82	No gender difference<br><br>High-strain (gender differences: p=0.83):	No differences according to follow-up duration (< *vs* ≥ 10 years)<br><br>The risk of CHD-associated high-strain	Multivariate-adjusted	Fixed effects model when no statistical heterogeneity	Yes<br>No evidence of publication bias by visual	Yes<br>Newcastle-Ottawa Quality Assessment Scale

First author	Publication date	Ref. number	Gender differences	Results from other subgroup/sensitivity analyses	Adjustment variables of main results	Statistical methods for pooled estimates	Publication bias assessment	Quality assessment of primary studies and/or evidence
			--- Men: 1.21 (1.04-1.40)<br>--- Women: 1.14 (0.69-1.87)<br><br>Passive job (gender differences: p=0.92):<br>--- Men: 1.10 (0.91-1.34)<br>--- Women: 1.09 (0.87-1.36)<br><br>Active job (gender differences: p=0.81):<br>--- Men: 1.07 (0.93-1.24)<br>--- Women: 1.11 (0.84-1.48)<br><br>Low control (gender differences: p=0.49):<br>--- Men: 1.01 (0.93-1.10)<br>--- Women: 1.14 (0.81-1.60)<br><br>High demands (gender differences: p=0.38):<br>--- Men: 1.12 (0.94-1.33)<br>--- Women: 1.02 (0.90-1.14)	jobs was increased in studies with adequate adjustment of confounders (RR 1.18, 95% CI 1.04–1.34) and was much more pronounced in those with inadequate adjustment (RR 1.66, 95% CI 1.27–2.16).<br>Adequate adjustment was defined as adjustment of at least 5 of 7 factors among age, sex, diabetes mellitus or other measure of blood glucose, BMI or other measure of overweight/obesity, cholesterol, smoking, and hypertension/blood pressure.<br>Sensitivity analyses confirmed that the results were not influenced by the use of fixed-effect or random-effects model, or ORs rather than RRs		between studies ($P>0.10$ and $I^2<50\%$), otherwise random-effects model	inspection of the funnel plots or by using Egger's test	All studies were graded as good quality
Yang	2019	83	Subgroup analyses according to gender (any cancer):<br>Men: 1.25 (1.14–1.37), $I^2=7.3\%$<br>Women: 1.03 (0.92–1.16), $I^2= 0.0\%$	Subgroup analyses according to study design, study location, cancer site, smoking, drinking, body mass index, and physical activity	Any adjustment	Random effects model	No	Yes<br>Newcastle-Ottawa Quality Assessment Scale
Yang	2018	84		For those meta-analyses showing between-studies heterogeneity, subgroup analyses were performed and showed differences according to study design:<br><br>--- effort-reward balance<br>prospective (2 studies):<br>1.93 (1.48-2.52)<br>cross-sectional (3 studies):<br>1.35 (1.28-1.43)<br>p-value for difference=0.02<br><br>--- work-family conflict<br>prospective (6 studies):		Fixed or random effects model according to heterogeneity between studies	Yes<br>Based on Begg's test, no publication was detected, although certain asymmetrical funnel plots were observed. Egger's test indicated that publication bias may be apparent for effort-reward	No

First author	Publication date	Ref. number	Gender differences	Results from other subgroup/sensitivity analyses	Adjustment variables of main results	Statistical methods for pooled estimates	Publication bias assessment	Quality assessment of primary studies and/or evidence
				1.83 (1.51-2.21)<br>cross-sectional (2 studies):<br>4.88 (3.59-6.64)<br>p-value for difference<0.01<br><br>No difference according to method of outcome measurement (standard scale *vs* nonstandard questionnaire) and study location (Europe *vs* Asia)			imbalance (p=0.04), low social support (p=0.02), and work-family conflict (p=0.03)	
Zhu	2020	85	--- Men: 1.17 (1.05-1.32), heterogeneity: I^2=68.9%<br>--- Women: 1.11 (1.01-1.21), heterogeneity: I^2=61.8%<br>No gender difference	No difference according to study design<br>Cohort (8 studies): 1.12 (1.00-1.25), heterogeneity: I^2=71.0%<br>Cross-sectional (21 studies):<br>1.14 (1.06-1.21), heterogeneity: I^2=77.6%<br><br>No difference according to outcome<br>BMI ≥ 25 kg/m^2: 1.07 (1.00-1.14), heterogeneity: I^2=69.1%<br>BMI ≥ 30 kg/m^2: 1.23 (1.09-1.39), heterogeneity: I^2=80.8%<br>Weight gain/BMI increase:<br>1.19 (1.02-1.40), heterogeneity: I^2=74.8%<br><br>No differences according to location, occupation, exposure definition, and study quality<br><br>Similar results when including only the results of studies whose participants excluded part-time workers (defined as <35 hours per week): 1.13 (1.04-1.22)<br>Excluding 5 studies that did not adjust for any confounding factors did not substantially alter results:<br>1.15 (1.08-1.23)	None or any adjustment	Random effects model	Yes<br>Visual inspection of the funnel plot showed asymmetry. Begg's test (p=0.035) and Egger's test (p<0.001) suggested that there existed significant evidence of potential publication bias	Yes<br>National Institutes of Health (NIH)'s Quality Assessment Tool for Observational Cohort and Cross-Sectional Studies

IPD-Work consortium studies with literature review were underlined in orange and those without literature review were underlined in yellow

Supplementary Table S2. Quality assessment of the included reviews

First author	Journal	Publication date	Reference number	Quality assessment of reviews (see Appendix 2)								Overall score for quality assessment of reviews (/16)
Amiri	PH	2020	14	●	●	○	⭘	●	⭘	⭘	●	11
Aronsson<br>Not in the extraction	BMCPH	2017	15	●	⭘	⭘	⭘	⭘	○	○	○	6
Babu	OEM	2014	16	●	⭘	○	⭘	●	○	⭘	⭘	8
Bonde	SJWEH	2013	17	⭘	○	○	⭘	⭘	⭘	⭘	⭘	6
Bonzini	OEM	2007	18	⭘	○	○	⭘	●	⭘	⭘	⭘	7
Cai	AJOG	2019	19	●	○	○	●	●	⭘	⭘	●	10
Cosgrove	OM	2012	20	●	○	○	⭘	●	○	⭘	○	6
Descatha	EI	2020	21	●	⭘	⭘	⭘	●	●	●	●	13
Dragano	E	2017	22	∅	●	●	∅	●	●	●	∅	16*
Duchaine	JAMAP	2020	23	●	●	⭘	⭘	●	○	⭘	●	11
Ferrie	CMAJ	2016	24	∅	●	●	∅	●	●	●	∅	16*
Fransson	AJE	2012	25	∅	●	⭘	∅	●	●	⭘	∅	12.8*
Fransson	S	2015	26	∅	●	⭘	∅	●	○	●	∅	11.2*
Hauke	WS	2011	27	⭘	●	○	⭘	●	○	○	○	6
Heikkila<br>Not in the extraction	BJC	2016	28	∅	●	⭘	∅	●	○	●	∅	11.2*
Heikkila	BMJ	2013	29	∅	●	⭘	∅	●	○	●	∅	11.2*
Heikkila	JAHA	2020	30	∅	●	⭘	∅	●	●	●	∅	14.4*
Heikkila	PO	2012	31	∅	●	●	∅	●	●	⭘	∅	14.4*
Heikkila	PO	2012	32	∅	●	●	∅	●	●	⭘	∅	14.4*
Heikkila	PO	2014	33	∅	●	⭘	∅	●	○	●	∅	11.2*
Huang	N	2015	34	●	●	○	⭘	●	●	⭘	●	12
Kang	JOEM	2012	35	⭘	⭘	○	○	●	○	●	●	8
Kim	IAOEH	2016	36	●	●	○	○	●	○	⭘	●	9
Kivimaki	CMAJ	2013	37	∅	●	⭘	∅	●	○	○	∅	8*
Kivimaki<br>Update of Kivimaki E 2014	CCR	2015	38	⭘	●	○	○	○	○	○	○	3
Kivimaki<br>Idem Steptoe ARPH 2013	E	2014	39	⭘	●	○	○	○	○	●	○	5
Kivimaki<br>Not in the extraction	E	2018	40	∅	●	●	∅	●	○	○	∅	9.6*
Kivimaki	EHJ	2017	41	∅	●	●	∅	●	●	●	∅	16*
Kivimaki	IJO	2015	42	●	●	●	○	●	○	⭘	○	9
Kivimaki	L	2012	43	∅	●	●	∅	●	●	●	∅	16*
Kivimaki	L	2015	44	●	●	●	⭘	●	●	●	●	15
Kivimaki	LDE	2015	45	●	●	⭘	○	●	●	⭘	●	12
Kivimaki	SJWEH	2006	46	⭘	●	⭘	○	●	○	⭘	⭘	7
Kraatz	IAOEH	2013	47	●	●	○	⭘	●	○	⭘	○	8
Lang	SSM	2012	48	⭘	●	○	⭘	●	○	⭘	⭘	8
Li	EI	2020	49	●	⭘	⭘	⭘	●	●	●	●	13
Linton<br>Not in the extraction	SMR	2015	50	●	⭘	○	⭘	●	○	○	○	6

First author	Journal	Publication date	Reference number	Quality assessment of reviews (see Appendix 2)								Overall score for quality assessment of reviews (/16)
Madsen	PM	2017	51	●	●	⭘	⭘	●	●	●	○	12
Magnavita	FN	2019	52	●	○	○	⭘	●	○	⭘	⭘	7
Milner	OEM	2018	53	●	○	○	○	●	⭘	⭘	⭘	7
Milner	JECH	2019	54	●	⭘	○	⭘	●	●	⭘	⭘	10
Nielsen	SMR	2020	55	●	○	○	⭘	○	⭘	⭘	●	7
Nieuwenhuijsen	OM	2010	56	⭘	●	○	⭘	⭘	⭘	○	○	6
Nyberg	DC	2014	57	∅	●	●	∅	⭘	●	●	∅	14.4*
Nyberg	JIM	2012	58	∅	●	●	∅	⭘	●	○	∅	11.2*
Nyberg	PO	2013	59	∅	○	●	∅	⭘	⭘	○	∅	6.4*
Palmer<br>Not in the extraction	OEM	2013	60	⭘	○	○	⭘	●	⭘	○	⭘	6
Rönnblad	SJWEH	2019	61	●	●	●	⭘	●	○	⭘	●	12
Rudkjoebing	SJWEH	2020	62	●	○	●	⭘	●	○	⭘	●	10
Rugulies	SJWEH	2017	63	●	●	○	⭘	●	○	⭘	⭘	9
Stansfeld	SJWEH	2006	64	●	●	○	○	●	⭘	⭘	○	8
Steptoe<br>Not in the extraction<br>Update of Kivimäki SJWEH 2006	NRC	2012	65	⭘	●	⭘	○	●	○	●	○	8
Sui	PO	2016	66	●	●	○	⭘	●	⭘	⭘	●	11
Szerencsi	SJWEH	2012	67	⭘	○	○	⭘	●	○	○	⭘	5
Taouk	SJWEH	2020	68	●	●	○	⭘	●	●	⭘	⭘	11
Theorell<br>Not in the extraction	BMCPH	2015	69	⭘	⭘	⭘	⭘	⭘	○	○	●	7
van der Molen<br>Update of Nieuwenhuijsen OM 2010	BMJO	2020	70	●	●	○	●	●	⭘	●	●	13
Van Melick	IAOEH	2014	71	●	○	○	⭘	●	⭘	⭘	○	7
Virtanen	AJE	2012	72	●	○	⭘	○	●	○	⭘	●	8
Virtanen	BMJ	2013	73	●	●	⭘	○	●	●	●	○	11
Virtanen	BMJ	2015	74	●	●	○	○	●	●	⭘	●	11
Virtanen<br>Update of Kivimäki L 2015	CCR	2018	75	●	●	●	○	●	○	●	○	10
Virtanen<br>Not in the extraction	IJE	2005	76	⭘	○	○	○	●	○	⭘	○	4
Virtanen	IJO	2020	77	∅	●	●	∅	●	●	⭘	∅	14.4*
Virtanen	SJWEH	2018	78	●	●	○	⭘	●	●	●	●	13
Watanabe	OEM	2016	79	●	●	○	○	●	●	●	⭘	11
Watanabe	OR	2018	80	●	●	○	⭘	●	○	○	○	7
Wong	IJERPH	2019	81	⭘	○	○	○	●	●	○	○	5
Xu	AM	2015	82	●	●	○	⭘	●	●	⭘	●	12
Yang	IJC	2019	83	●	⭘	○	⭘	●	●	●	○	10
Yang	SB	2018	84	●	○	○	○	●	○	⭘	⭘	6
Zhu	OR	2020	85	●	○	○	⭘	●	●	⭘	⭘	9

IPD-Work consortium studies with literature review were underlined in orange and those without literature review were underlined in yellow

Supplementary Table S3. Full-text articles excluded and reason for exclusion

**Full-text articles excluded (first author, publication year, ref number)**	**Reason for exclusion**
Alarcon 2011 (1)	3
Cantisano 2008 (2)	2
Chan 2008(3)	2
Cheng 2008 (4)	2
Crawford 2010 (5)	3
Eddy 2016 (6)	3
Eddy 2017 (7)	3
Eddy 2018 (8)	3
Eijckelhof 2013 (9)	2
Heikkilä 2013 (10)	2
Jiang 2018 (11)	3
Kivimäki 2015 (12)	1
Landsbergis 2013 (13)	3
Litwiller 2017 (14)	3
Llosa 2018 (15)	3
Montano 2017 (16)	3
Nielsen 2012 (17)	3
Nielsen 2014 (18)	2
Nielsen 2015 (19)	3
Nixon 2011 (20)	3
Park 2009 (21)	3
Park 2016 (22)	3
Reichl 2014 (23)	3
Schmidt 2014 (24)	3
Sverke 2002 (25)	2
Verkuil 2015 (26)	3
Willness 2007 (27)	3

1. neither a literature review, nor a IPD-Work study (n = 1)
2. no or unspecified health outcome (n = 7)
3. RR, HR or OR summary estimates not provided (n = 19)

References

1. Alarcon GM. A meta-analysis of burnout with job demands, resources, and attitudes. Journal of Vocational Behavior. 2011;79(2):549-62.
2. Cantisano GT, Domínguez JFM, Depolo M, Howard VN. Perceived sexual harassment at work: Meta-analysis and structural model of antecedents and consequences. The Spanish Journal of Psychology. 2008;11(1):207-18.
3. Chan DK-S, Lam CB, Chow SY, Cheung SF. Examining the Job-Related, Psychological, and Physical Outcomes of Workplace Sexual Harassment: A Meta-Analytic Review. Psychology of Women Quarterly. 2008;32(4):362-76.
4. Cheng GH-L, Chan DK-S. Who suffers more from job insecurity? A meta-analytic review. Applied Psychology. 2008;57(2):272-303.

5. Crawford ER, LePine JA, Rich BL. Linking Job Demands and Resources to Employee Engagement and Burnout: A Theoretical Extension and Meta-Analytic Test. Journal of Applied Psychology. 2010;95(5):834-48.
6. Eddy P, Heckenberg R, Wertheim EH, Kent S, Wright BJ. A systematic review and meta-analysis of the effort-reward imbalance model of workplace stress with indicators of immune function. Journal of Psychosomatic Research. 2016;91:1-8.
7. Eddy P, Wertheim EH, Kingsley M, Wright BJ. Associations between the effort-reward imbalance model of workplace stress and indices of cardiovascular health: A systematic review and meta-analysis. Neurosci Biobehav Rev. 2017;83:252-66.
8. Eddy P, Wertheim EH, Hale MW, Wright BJ. A Systematic Review and Meta-analysis of the Effort-Reward Imbalance Model of Workplace Stress and Hypothalamic-Pituitary-Adrenal Axis Measures of Stress. Psychosom Med. 2018;80(1):103-13.
9. Eijckelhof BHW, Huysmans MA, Garza JLB, Blatter BM, van Dieen JH, Dennerlein JT, et al. The effects of workplace stressors on muscle activity in the neck-shoulder and forearm muscles during computer work: a systematic review and meta-analysis. European Journal of Applied Physiology. 2013;113(12):2897-912.
10. Heikkila K, Fransson EI, Nyberg ST, Zins M, Westerlund H, Westerholm P, et al. Job strain and health-related lifestyle: findings from an individual-participant meta-analysis of 118,000 working adults. Am J Public Health. 2013;103(11):2090-7.
11. Jiang L, Lavaysse LM. Cognitive and Affective Job Insecurity: A Meta-Analysis and a Primary Study. Journal of Management. 2018;44(6):2307-42.
12. Kivimaki M, Singh-Manoux A, Virtanen M, Ferrie JE, Batty GD, Rugulies R. IPD-Work consortium: pre-defined meta-analyses of individual-participant data strengthen evidence base for a link between psychosocial factors and health. Scand J Work Environ Health. 2015;41(3):312-21.
13. Landsbergis PA, Dobson M, Koutsouras G, Schnall P. Job strain and ambulatory blood pressure: a meta-analysis and systematic review. Am J Public Health. 2013;103(3):e61-71.
14. Litwiller B, Snyder LA, Taylor WD, Steele LM. The relationship between sleep and work: A meta-analysis. The Journal of Applied Psychology. 2017;102(4):682-99.
15. Llosa JA, Menendez-Espina S, Agullo-Tomas E, Rodriguez-Suarez J. Job insecurity and mental health: A meta-analytical review of the consequences of precarious work in clinical disorders. Anales De Psicologia. 2018;34(2):211-23.
16. Montano D, Reeske A, Franke F, Huffmeier J. Leadership, followers' mental health and job performance in organizations: A comprehensive meta-analysis from an occupational health perspective. Journal of Organizational Behavior. 2017;38(3):327-50.
17. Nielsen MB, Einarsen S. Outcomes of exposure to workplace bullying: A meta-analytic review. Work and Stress. 2012;26(4):309-32.
18. Nielsen MB, Magerøy N, Gjerstad J, Einarsen S. Workplace bullying and subsequent health problems. Tidsskr Nor Laegeforen. 2014;134(12-13):1233-8.
19. Nielsen MB, Tangen T, Idsoe T, Matthiesen SB, Magerøy N. Post-traumatic stress disorder as a consequence of bullying at work and at school A literature review and meta-analysis. Aggression and Violent Behavior. 2015;21:17-24.
20. Nixon AE, Mazzola JJ, Bauer J, Krueger JR, Spector PE. Can work make you sick? A meta-analysis of the relationships between job stressors and physical symptoms. Work and Stress. 2011;25(1):1-22.
21. Park HI, Baiden M, Jacob AC, Wagner SH, editors. Job control and burnout: A meta-analytic test of the Conservation of Resources model2009 2009: Academy of Management (Acad. Manage. Annu. Meet.: Green Manage. Matters, AOM).

22. Park HI, Jacob AC, Wagner SH, Baiden M. Job control and burnout: A meta-analytic test of the conservation of resources model. Applied Psychology. 2016;63(4):607-42.
23. Reichl C, Leiter MP, Spinath FM. Work-nonwork conflict and burnout: A meta-analysis. Human Relations. 2014;67(8):979-1005.
24. Schmidt S, Roesler U, Kusserow T, Rau R. Uncertainty in the workplace: Examining role ambiguity and role conflict, and their link to depression—a meta-analysis. European Journal of Work and Organizational Psychology. 2014;23(1):91-106.
25. Sverke M, Hellgren J, Naswall K. No security: a meta-analysis and review of job insecurity and its consequences. J Occup Health Psychol. 2002;7(3):242-64.
26. Verkuil B, Atasayi S, Molendijk ML. Workplace Bullying and Mental Health: A Meta-Analysis on Cross-Sectional and Longitudinal Data. PLoS One. 2015;10(8):e0135225.
27. Willness CR, Steel P, Lee K. A meta-analysis of the antecedents and consequences of workplace sexual harassment. Personnel Psychology. 2007;60(1):127-62.
